# Clopidogrel vs. prasugrel vs. ticagrelor in patients with acute myocardial infarction complicated by cardiogenic shock: a pooled IABP-SHOCK II and CULPRIT-SHOCK trial sub-analysis

**DOI:** 10.1007/s00392-021-01866-3

**Published:** 2021-05-17

**Authors:** Martin Orban, Jan Kleeberger, Taoufik Ouarrak, Anne Freund, Hans-Josef Feistritzer, Georg Fuernau, Tobias Geisler, Kurt Huber, Dariusz Dudek, Marko Noc, Gilles Montalescot, Alexander Neumer, Paul Haller, Peter Clemmensen, Uwe Zeymer, Steffen Desch, Steffen Massberg, Steffen Schneider, Holger Thiele, Jörg Hausleiter

**Affiliations:** 1 Department of Medicine I, University Hospital, Ludwig-Maximilians-University, Munich, Germany; 2 German Center for Cardiovascular Research (DZHK), Partner Site Munich, Munich Heart Alliance, Munich, Germany; 3 Stiftung Institut für Herzinfarktforschung, Ludwigshafen, Germany; 4 Heart Center Leipzig at University of Leipzig, Leipzig, Germany; 5 University Heart Center Lübeck, Lübeck, Germany; 6 University Hospital of Tübingen, Tübingen, Germany; 7 Medical Faculty, 3rd Department of Medicine, Cardiology and Intensive Care Medicine, Wilhelminenhospital, and Sigmund Freud University, Vienna, Austria; 8 Jagiellonian University, Krakow, Poland; 9 University Medical Center Ljubljana, Ljubljana, Slovenia; 10 Sorbonne Université, ACTION Group, Hôpital Pitié-Salpêtrière (AP-HP), Paris, France; 11 Universitäres Herz- Und Gefäßzentrum UKE Hamburg, Klinik Und Poliklinik Für Kardiologie, Hamburg, Germany; 12 Medizinische Klinik und Poliklinik I, LMU Klinikum München, Campus Großhadern, Ludwig-Maximilians-Universität München, Marchioninistr. 15, 81377 München, Germany

**Keywords:** Clopidogrel, Prasugrel, Ticagrelor, ADP-receptor antagonists, Cardiogenic shock, Bleeding

## Abstract

**Aims:**

The aim of this pooled sub-analysis of the Intraaortic Balloon Pump in Cardiogenic Shock II (IABP-SHOCK II) and Culprit Lesion Only PCI versus Multivessel PCI in Cardiogenic Shock (CULPRIT-SHOCK) trial was to compare the clinical outcome of patients with acute myocardial infarction complicated by cardiogenic shock treated either with clopidogrel or the newer, more potent ADP-receptor antagonists prasugrel or ticagrelor.

**Methods and results:**

For the current analysis the primary endpoint was 1-year mortality and the secondary safety endpoint was moderate or severe bleedings until hospital discharge with respect to three different ADP-receptor antagonists. 856 patients were eligible for analysis. Of these, 507 patients (59.2%) received clopidogrel, 178 patients (20.8%) prasugrel and 171 patients (20.0%) ticagrelor as acute antiplatelet therapy. The adjusted rate of mortality after 1-year did not differ significantly between prasugrel and clopidogrel (hazard ratio [HR]: 0.81, 95% confidence interval [CI] 0.60–1.09, p_adj_ = 0.17) or between ticagrelor and clopidogrel treated patients (HR: 0.86, 95% CI 0.65–1.15, p_adj_ = 0.31). In-hospital bleeding events were significantly less frequent in patients treated with ticagrelor vs. clopidogrel (HR: 0.37, 95% CI 0.20 -0.69, p_adj_ = 0.002) and not significantly different in patients treated with prasugrel vs. clopidogrel (HR: 0.73, 95% CI 0.43 -1.24, p_adj_ = 0.24).

**Conclusion:**

This pooled sub-analysis is the largest analysis on safety and efficacy of three oral ADP-receptor antagonists and shows that acute therapy with either clopidogrel, prasugrel or ticagrelor is no independent predictor of 1-year mortality. Treatment with ticagrelor seems independently associated with less in-hospital moderate and severe bleeding events compared to clopidogrel. This finding might be due to selection bias and should be interpreted with caution.

**Graphic abstract:**

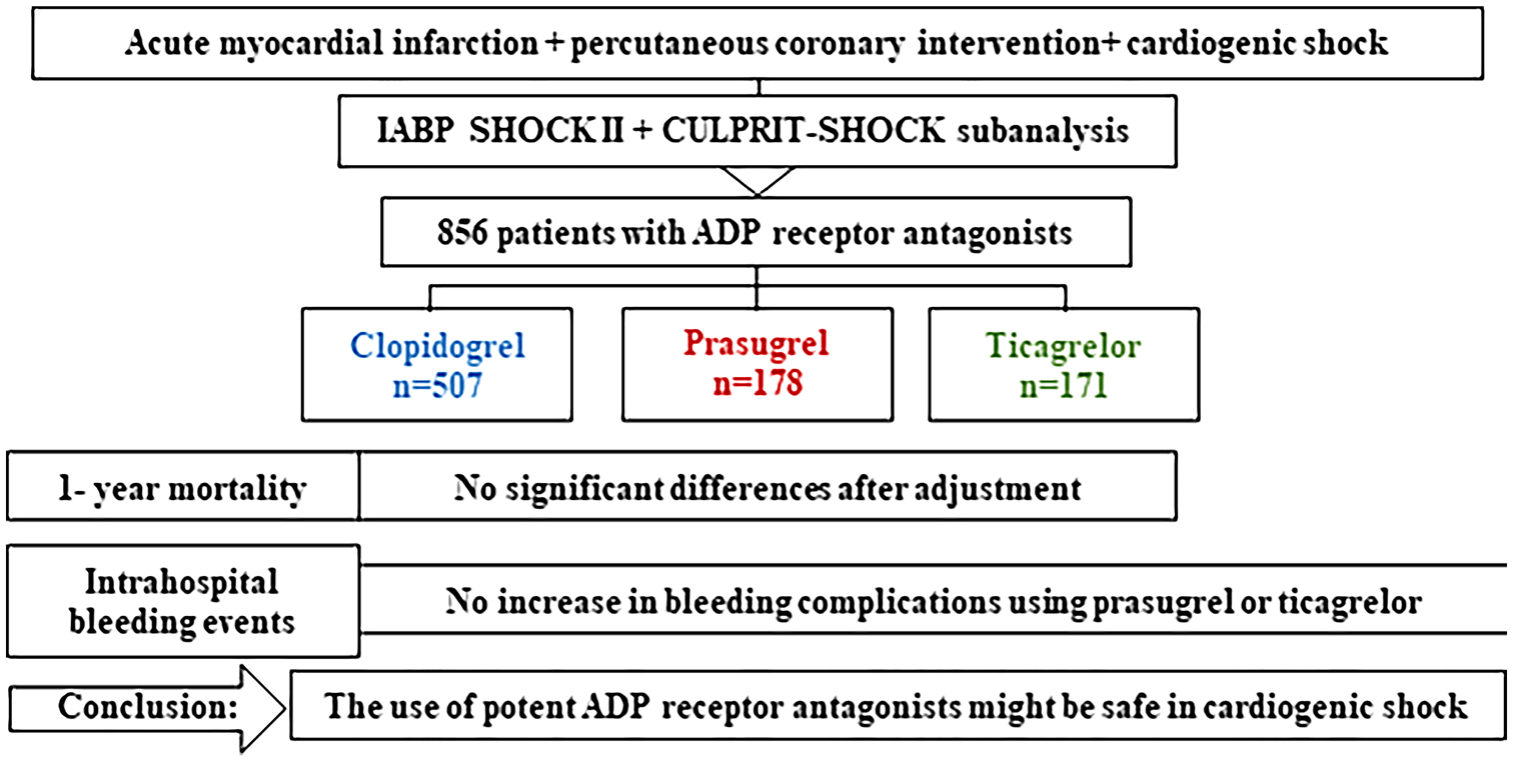

**Supplementary Information:**

The online version contains supplementary material available at 10.1007/s00392-021-01866-3.

## Introduction

An important complication of acute myocardial infarction (AMI) is cardiogenic shock affecting around 5–10% of all AMI cases. It considerably worsens the prognosis [1, 2]. Recent randomized trials report a 30-day mortality rate in the range of 40–52% in this entity [3]. Large registries even report mortality rates up to 70% at 1-year follow-up [2, 4]. As stated in the latest guidelines, primary percutaneous coronary intervention (PCI) is the standard reperfusion therapy for patients with AMI complicated by cardiogenic shock [5]. Subsequently, a dual antiplatelet therapy consisting of acetylsalicylic acid (ASA) and an adenosine-diphosphate (ADP)-receptor antagonist is indicated for patients treated by PCI [5]. The comparative safety, efficacy and antiplatelet action of different available ADP-receptor inhibitors in this patient cohort remain understudied, because the landmark trials comparing the more potent ADP-receptor inhibitors prasugrel and ticagrelor to clopidogrel excluded patients with cardiogenic shock [6, 7]. A sub-analysis of the Intraaortic Balloon Pump in Cardiogenic Shock II (IABP-SHOCK II) trial and accompanying registry indicated that the use of potent ADP-receptor antagonists—predominantly prasugrel—is feasible and might not be harmful in patients with cardiogenic shock complicating AMI [8, 9]. Noteworthy, the number of studied patients treated with ticagrelor was negligible in the latter analyses due to its late approval in 2011. Thus, the aim of this pooled sub-analysis of the IABP-SHOCK II and Culprit Lesion Only PCI versus Multivessel PCI in Cardiogenic Shock (CULPRIT-SHOCK) trial was to investigate the safety and efficacy of the more vigorously and rapidly acting oral ADP-receptor antagonists prasugrel and especially ticagrelor.

## Methods

### Patient selection

The trial designs and results of both the IABP-SHOCK II and the CULPRIT-SHOCK trial have been published previously [3, 10]. Briefly, both studies were prospective, randomized, open-label, multicentre, controlled trials in patients with acute ST-elevation myocardial infarction (STEMI) and non-STEMI complicated by cardiogenic shock with the intention to undergo early revascularization (PCI or alternatively bypass surgery [only in the IABP-SHOCK II trial], for complete inclusion and exclusion criteria see Thiele et al. [3, 10]). In total, 600 patients with AMI and cardiogenic shock were randomized to IABP or no IABP treatment in the IABP-SHOCK II trial. In the CULPRIT-SHOCK trial, 686 patients were randomly assigned to undergo either culprit-lesion-only PCI with possible staged revascularization or immediate multivessel PCI.

### Treatment with ADP-receptor antagonists

The use of ADP-receptor antagonists was left to the discretion of the treating physicians. There was neither a specific protocol nor randomization which drug to use or when and how to administer. The majority of the study patients were mechanically ventilated (53.9%) on admission and received the respective ADP-receptor antagonist via a naso-gastric tube to ensure enteral absorption.

### Study endpoints, inclusion and exclusion criteria

The primary endpoint of this pooled, non-randomized observational sub-analysis was the post-procedural 1-year mortality with respect to three different ADP-receptor antagonists (clopidogrel vs. prasugrel vs. ticagrelor). The secondary safety endpoint was moderate or severe bleedings according to GUSTO criteria [11] until hospital discharge with respect to all three ADP-receptor antagonists. Further endpoints comprised 30-day mortality, stroke, myocardial infarction, all bleeding complications and severe or life-threatening bleedings as assessed according to GUSTO criteria during 1-year follow-up in surviving patients. Inclusion and exclusion criteria were adapted from the previously published [8] sub-analysis of the IABP-SHOCK II trial on ADP-receptor antagonists and were the following: Patients receiving either clopidogrel, prasugrel or ticagrelor as acute medical therapy qualified for inclusion into this subgroup-analysis. Patients who died before PCI, patients not receiving any ADP-receptor antagonist as acute antiplatelet therapy, patients with no information on ADP-receptor antagonist treatment and patients receiving any combination of clopidogrel, prasugrel or ticagrelor simultaneously as acute antiplatelet therapy were excluded from analysis.

### Statistical analysis

Detailed information on statistical analysis can be found in the Supplement. The impact of acute medication on mortality and bleeding was examined in unadjusted and adjusted regression analyses, the corresponding odds ratios (OR) or hazard ratios (HR) with 95%-confidence intervals (CI) are presented. We included variables in both models that show an association with at least one of the two outcome variables (in-hospital bleeding or 1-year bleeding) by univariate analysis at *p* < 0.05. For the bleeding model, we used a logistic regression for the in-hospital events and a Cox proportional hazards regression for the bleeding complications until the end of follow-up. The following variables were entered in both multivariable models for bleeding complications: age and acute medication as fixed parameter, gender, previous myocardial infarction, resuscitation within 24 h before randomization, mechanical ventilation, creatinine on admission [µmol/l], lactate > 2 mmol/l on admission, treatment with unfractionated heparin and active mechanical circulatory support. Concerning the model for mortality, variables entered in the model were age, female, previous myocardial infarction, previous PCI, previous coronary artery bypass graft (CABG) surgery, previous stroke, known renal insufficiency (glomerular filtration rate, GFR < 30 ml/min), resuscitation within 24 h before randomization, ST-segment elevation, creatinine on admission [µmol/l], heart rate [bpm] before PCI, systolic blood pressure [mmHg] before PCI, SAPS II Score.

## Results

### Study population

**Fig. 1:**
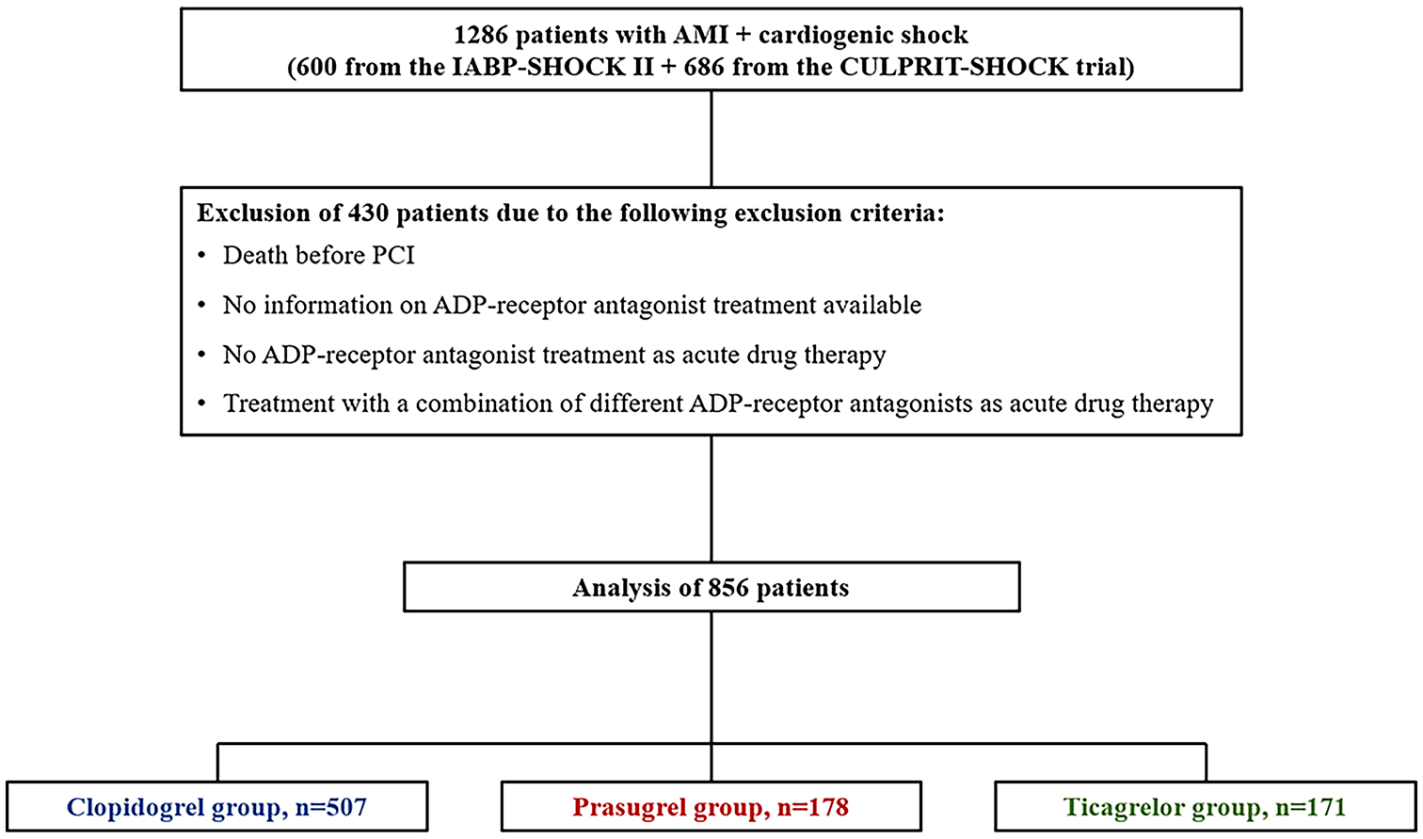
Study flow chart of the pooled sub-analysis on ADP-receptor antagonists in patients from the IABP-SHOCK II and CULPRIT-SHOCK trial. *AMI* acute myocardial infarction, *PCI* percutaneous coronary intervention

Information on patients who were excluded from the analysis according to the exclusion criteria mentioned above are displayed in Supplemental Tables 8, 9, 10, 11, 12, 13, 14, 15 and 16. After exclusion of 430 patients according to the exclusion criteria, 856 patients were analysed. Of these, 507 patients (59.2%) received clopidogrel, 178 patients (20.8%) prasugrel and 171 patients ticagrelor (20.0) as acute antiplatelet therapy (Fig. 1). The mean age of the clopidogrel (69 ± 12 years) and ticagrelor (69 ± 12 years) group was similar, but patients receiving prasugrel were significantly younger (62 ± 11 years). The essential baseline characteristics of all three subgroups are shown in Table 1; cf. Supplemental Table 2 for the extended baseline characteristics of the cohorts. Regarding the clinical presentation prior randomization during the primary trials, patients treated with prasugrel or ticagrelor were more often resuscitated before randomization (Prasugrel: *n* = 87/178, 48.9%; Ticagrelor: *n* = 85/171, 49.7%; Clopidogrel: *n* = 204/506, 40.3%, *p* = 0.034), presented more often with STEMI (Prasugrel: *n* = 143/176, 81.3%; Ticagrelor: *n* = 118/167, 70.7%; Clopidogrel: *n* = 296/506, 58.5%, *p* < 0.001) and with lower levels of creatinine (Prasugrel: median = 102.27 [IQR 86.32, 133.30] µmol/l; Ticagrelor: median = 108.86 [IQR 91.05, 136.0] µmol/l; Clopidogrel: median = 116.50 [IQR 96.0, 151.0] µmol/l, *p* < 0.001) (Supplemental Table 3) compared to clopidogrel. The treatment strategy did not differ between the cohorts with respect to the application of PCI, although bare-metal stents (BMS) were more frequently implanted in patients treated with clopidogrel (Prasugrel: *n* = 41/171, 24.0%; Ticagrelor: *n* = 13/162, 8.0%; Clopidogrel: *n* = 234/466, 50.2%, *p* < 0.001). Glycoprotein (GP) IIb/IIIa inhibitors were more frequently used within the prasugrel (*n* = 79/178, 44.4%) and clopidogrel group (*n* = 189/507, 37.3%) compared to the ticagrelor group (*n* = 44/171, 25.7%, *p* = 0.001). In addition, a higher number of clopidogrel-treated patients required mechanical ventilation (*n* = 407/507, 80.3%, *p* = 0.023) in comparison to prasugrel (*n* = 126/177, 71.2%) or ticagrelor (*n* = 126/171, 73.7%)-treated patients. There was no difference in the use of targeted temperature management between groups (Supplemental Table 3).

**Table 1:** Baseline characteristics of the study cohorts

Variable	Clopidogrel *n* = 507	Prasugrel *n* = 178	Ticagrelor *n* = 171	*P* value
Age, years ± SD	69 ± 12	62 ± 11	69 ± 12	< 0.001
Female gender, *n* (%)	160 (31.6)	41(23.0)	42 (24.6)	0.044
Body-mass index*, median (IQR)	27.2 (24.5, 30.1)	26.3 (24.2, 28.9)	27.7 (24.7, 29.4)	0.77
Cardiovascular risk factors – no./total no. (%)				
Current smoking	144/501 (28.7)	80/176 (45.5)	46/167 (27.5)	< 0.001
Hypertension	353/504 (70)	112/178 (62.9)	92/170 (54.1)	< 0.001
Hypercholesterolemia	187/503 (37.2)	67/178 (37.6)	55/169 (32.5)	0.51
Diabetes mellitus	180/505 (35.6)	35/178 (19.7)	51/169 (30.2)	< 0.001
Morbidities				
Prior myocardial infarction, no./total no. (%)	121/506 (23.9)	35/178 (19.7)	19/170 (11.2)	0.002
Prior stroke, no./total no. (%)	51/506 (10.1)	3/178 (1.7)	9/169 (5.3)	< 0.001
Prior PCI, no./total no. (%)	108/506 (21.3)	39/178 (21.9)	22/170 (12.9)	0.043
Renal impairment (GFR < 30 ml/min), no./total no. (%)	106/506 (20.9)	13/178 (7.3)	13/169 (7.7)	< 0.001
Chronic drug therapy, no./total no. (%)				
ASA	210/473 (44.4)	50/163 (30.7)	51/140 (36.4)	0.005
Clopidogrel	72/474 (15.2)	10/162 (6.2)	6/135 (4.4)	< 0.001
Prasugrel	1/474 (0.2)	3/162 (1.9)	0/135 (0.0)	0.028
Ticagrelor	2/358 (0.6)	1/137 (0.7)	15/136 (11.0)	< 0.001
Vitamin K-antagonists	31/473 (6.6)	4/161 (2.5)	3/134 (2.2)	0.034

This table shows baseline characteristics of the clopidogrel, the prasugrel and the ticagrelor subgroup. Data presented are means (± standard deviation, SD), medians [interquartile range, IQR] or numbers of patients (percentages)

*Body-mass index = weight in kilograms divided by the square of the height in meters

*PCI* percutaneous coronary intervention, *CABG* coronary artery bypass graft, *GFR* glomerular filtration rate, *ASA* acetylsalicylic acid, *p*-values: Pearson chi-squared test or Mann–Whitney–Wilcoxon test

### Administration of clopidogrel, prasugrel and ticagrelor in IABP-SHOCK II vs. CULPRIT SHOCK

In IABP SHOCK II 77.9% (*n* = 387) of patients were treated with clopidogrel, 19% (*n* = 93) with prasugrel and 3.4% (*n* = 17) with ticagrelor. In CULPRIT-SHOCK 33.4% (*n* = 120) of patients were treated with clopidogrel, 24% (*n* = 85) with prasugrel and 43% (*n* = 154) with ticagrelor (*p* < 0.0001, Supplemental Table 1).

### Clinical outcome

**Fig. 2:**
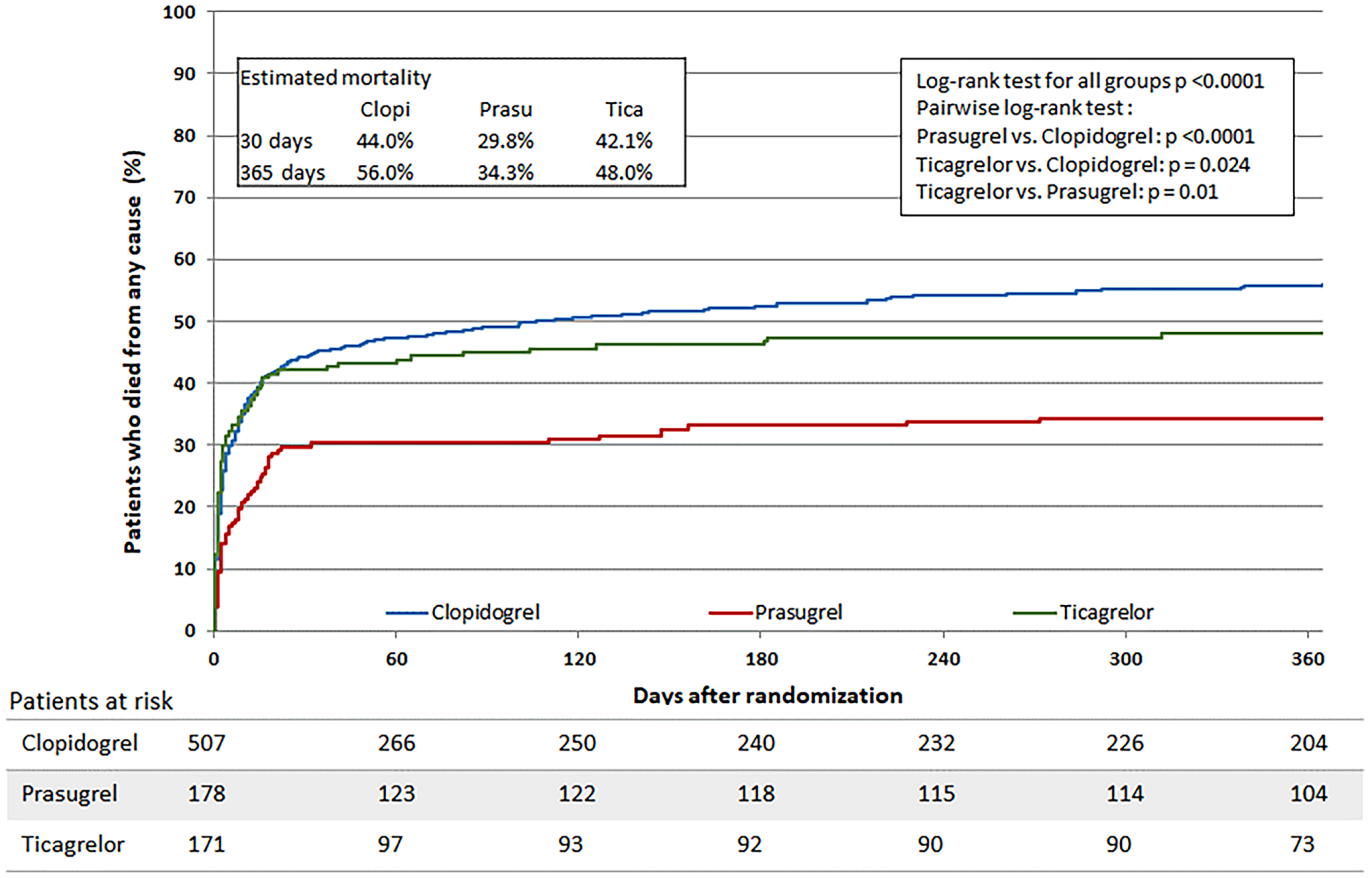
The Figure shows the survival curve during 1-year follow-up period in patients treated with clopidogrel (blue line), prasugrel (red line) or ticagrelor (green line)

The unadjusted rates of mortality at 1-year follow-up, as well as ischemic and bleeding events of all three subgroups at 30-days follow-up are listed in Table 2. The unadjusted all-cause 1-year mortality was lowest in prasugrel, followed by ticagrelor and highest in clopidogrel treated patients (34.9% [61/175] vs. 48.0% [82/171] vs. 55.9% [283/506] of patients, *p* < 0.001), Fig. 2. There was also a lower unadjusted all-cause 30-day mortality in prasugrel, followed by ticagrelor and highest in clopidogrel treated patients (29.8% [53/178] vs. 42.1% [72/171] vs. 43.9% [222/506], p < 0.01). The incidence of repeat myocardial infarction and ischemic stroke did not differ significantly between all three subgroups. Table 3 depicts a multivariate Cox regression model for 30-day and 1-year mortality. No significant differences were observed concerning the mortality risk with respect to the ADP-receptor antagonist applied. For 1-year mortality the adjusted hazard ratio was 0.81 (95% CI 0.60–1.09, *p* = 0.17) for patients treated with prasugrel vs. clopidogrel and 0.86 (95% CI 0.65–1.15, *p* = 0.31) for patients treated with ticagrelor vs. clopidogrel (see also Tables 4, 5).

**Table 2:** Clinical outcome at follow-up

Events, no./total no. (%)	Clopidogrel *n* = 507	Prasugrel *n* = 178	Ticagrelor *n* = 171	*P* value
30-days events				
Death ≤ 30 days	222/506 (43.9)	53/178 (29.8)	72/171 (42.1)	0.004
Postprocedural death ≤ 30 days	206/490 (42.0)	51/176 (29.0)	67/166 (40.4)	0.009
Renal replacement therapy ≤ 30 days	99/507 (19.5)	29/178 (16.3)	20/171 (11.7)	0.060
Events by survivors (≤ 30 days)				
Myocardial infarction ≤ 30 days	8/284 (2.8)	1/125 (0.8)	1/99 (1.0)	0.30
Stroke ≤ 30 days	2/284 (0.7)	5/125 (4.0)	2/99 (2.0)	0.065
PCI ≤ 30 days	17/284 (6.0)	10/125 (8.0)	4/99 (4.0)	0.47
1-year events				
Death ≤ 365 days	283/506 (55.9)	61/175 (34.9)	82/171 (48.0)	< 0.001
Postprocedural death ≤ 365 days	267/490 (54.5)	59/173 (34.1)	77/166 (46.4)	< 0.001
Events by survivors (≤ 365 days)				
Myocardial infarction ≤ 365 days	13/223 (5.8)	6/114 (5.3)	2/89 (2.2)	0.41
Stroke ≤ 365 days	5/223 (2.2)	5/114 (4.4)	3/89 (3.4)	0.55
PCI ≤ 365 days	46/223 (20.6)	35/114 (30.7)	24/89 (27.0)	0.11
Moderate and severe bleeding ≤ 365	36/223 (16.1)	14/114 (12.3)	6/89 (6.7)	0.081
Severe bleeding ≤ 365 days	5/223 (2.2)	6/114 (5.3)	0/89 (0.0)	0.057
Moderate bleeding ≤ 365 days	34/223 (15.2)	9/114 (7.9)	6/89 (6.7)	0.039
In-hospital bleeding events	94/507 (18.5)	25/177 (14.1)	17/171 (9.9)	0.022
Severe/life-threatening	18/507 (3.6)	12/177 (6.8)	7/171 (4.1)	0.19
Moderate	83/507 (16.4)	16/177 (9.0)	11/171 (6.4)	< 0.001

This table shows unadjusted death, ischemic and bleeding events of all three cohorts at 30 days and at 1-year follow-up. Data presented are numbers of patients (percentages). *PCI* percutaneous coronary intervention. *p*-values: Pearson chi-squared test or Mann–Whitney–Wilcoxon test

**Table 3:** Cox regression model of post-procedural 30- day and 1- year mortality

30-day mortality	Unadjusted OR (95% CI)	*P* value	Adjusted OR (95% CI)	*P* value
Prasugrel vs. Clopidogrel	0.62 (0.46–0.84)	0.0022	0.88 (0.63–1.22)	0.43
Ticagrelor vs. Clopidogrel	0.96 (0.73–1.26)	0.76	0.91 (0.66–1.26)	0.57

1-year mortality	Unadjusted OR (95% CI)	*P* value	Adjusted OR (95% CI)	*P* value
Prasugrel vs. Clopidogrel	0.54 (0.41–0.72)	< 0.0001	0.81 (0.60–1.09)	0.17
Ticagrelor vs. Clopidogrel	0.83 (0.65–1.07)	0.16	0.86 (0.65–1.15)	0.31

This table shows the multivariable Cox regression model of post-procedural 30- day and 1-year mortality with the different ADP-receptor inhibitors as dependent variable in comparison to clopidogrel. Adjusted odd ratios were calculated with an adjustment for the variables shown in the methods section. *P*-values: Pearson chi-squared test or Mann–Whitney–Wilcoxon test

**Table 4:** Regression analysis of bleeding events

Hospital bleeding events (moderate & severe)	Unadjusted OR (95% CI)	*P* value	Adjusted OR (95% CI)	*P* value
Prasugrel vs. Clopidogrel	0.72 (0.45–1.17)	0.18	0.73 (0.43–1.24)	0.24
Ticagrelor vs. Clopidogrel	0.49 (0.28–0.84)	0.01	0.37 (0.20–0.69)	0.002

1—year bleeding events (moderate & severe)	Unadjusted HR (95% CI)	*P* value	Adjusted HR (95% CI)	*P* value
Prasugrel vs. Clopidogrel	0.74 (0.49–1.11)	0.14	0.83 (0.54–1.28)	0.40
Ticagrelor vs. Clopidogrel	0.50 (0.30–0.83)	0.007	0.43 (0.25–0.72)	0.002

This table shows the regression analyses of bleeding events with the different ADP-receptor inhibitors as dependent variable in comparison to clopidogrel. Adjusted odds and hazard ratios were calculated with an adjustment for the variables shown in the methods section. *P*-values: Pearson chi-squared test or Mann–Whitney–Wilcoxon test

**Table 5:** Cox regression model of 30- day/ 1- year mortality and in- hospital/ 1-year bleeding events comparing Prasugrel vs. Ticagrelor

		Unadjusted HR (95%CI)	*p*-value	Adjusted HR (95%CI)	*p*-value
Endpoints					
Post-procedural 30-day mortality	Ticagrelor vs. Prasugrel	1.57 (1.09–2.26)	0.016	1.07 (0.72–1.61)	0.73
	Prasugrel vs. Clopidogrel/Ticagrelor	0.58 (0.43–0.77)	< 0.001	0.96 (0.70–1.30)	0.77
	Ticagrelor vs. Clopidogrel/Prasugrel	0.96 (0.74–1.24)	0.75	0.87 (0.65–1.18)	0.37
Post-procedural 365-day mortality	Ticagrelor vs. Prasugrel	1.56 (1.11–2.18)	0.01	1.09 (0.75–1.58)	0.65
	Prasugrel vs. Clopidogrel/Ticagrelor	0.56 (0.43–0.72)	< 0.0001	0.89 (0.67–1.17)	0.40
In- hospital severe/moderate bleeding	Ticagrelor vs. Prasugrel	0.67 (0.35–1.29)	0.23	0.51 (0.25–1.05)	0.068
	Prasugrel vs. Clopidogrel/Ticagrelor	0.79 (0.51–1.25)	0.31	0.83 (0.51–1.34)	0.44
	Ticagrelor vs. Clopidogrel/Prasugrel	0.51 (0.30–0.86)	0.01	0.46 (0.26–0.81)	0.006
Severe /moderate bleeding 365- days	Ticagrelor vs. Prasugrel	0.68 (0.38–1.22)	0.20	0.51 (0.28–0.95)	0.033
	Prasugrel vs. Clopidogrel/Ticagrelor	0.76 (0.52–1.12)	0.17	0.91 (0.61–1.36)	0.65
	Ticagrelor vs. Clopidogrel/Prasugrel	0.50 (0.31–0.81)	0.005	0.46 (0.28–0.75)	0.002

This table shows the unadjusted and adjusted Cox regression model of 30- day and 1- year mortality, as well as in- hospital and 1- year bleeding events comparing Prasugrel versus Ticagrelor; an unadjusted and adjusted regression analyses and corresponding odds ratio (OR) or hazard ratio (HR) with 95%-confidence interval (CI) are presented. All variables with *p*-value < 0.05 from the univariate comparison were entered in the multivariable models. For the bleeding model, a logistic regression for the in-hospital events and a Cox proportional hazards regression for the bleeding complications until the end of follow-up was used. Adjusted odd ratios for mortality were calculated with an adjustment for the following variables: Age, gender, previous myocardial infarction, known chronic kidney diseases (eGFR < 30 ml/min), previous PCI, previous CABG surgery, previous stroke, ST-segment elevation, creatinine on admission [µmol/l], heart rate before PCI, systolic blood pressure before PCI, SAPS II and resuscitation within 24 h before randomization. Adjusted odds and hazard ratios for bleeding events were calculated with an adjustment for the following variables: Age, gender, previous myocardial infarction, Creatinine on Admission [µmol/l], mechanical ventilation, resuscitation within 24 h before randomization, unfractionated heparin as acute drug therapy, serum lactate > 2 mmol/l on admission and active assist devices. *HR* hazard ratio, *OR* odds ratio, *CI* confidence interval, *eGFR* estimated glomerular filtration rate, *PCI* percutaneous coronary intervention, *CABG* coronary artery bypass graft, *SAPS II* simplified acute physiology score II. *P*-values: Pearson chi-squared test or Mann–Whitney–Wilcoxon test

**Fig. 3:**
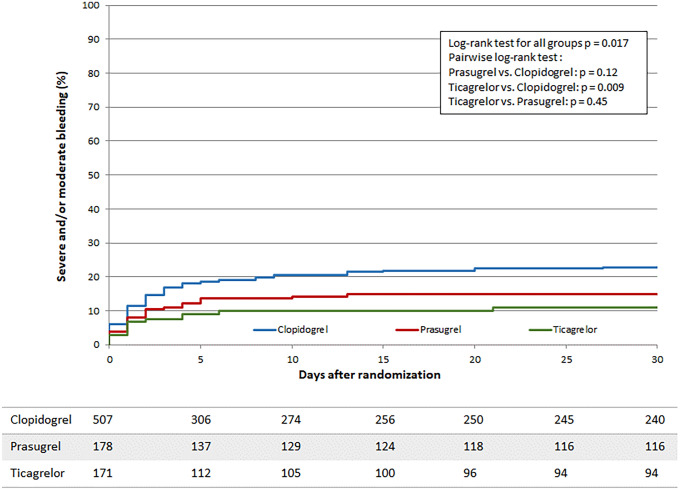
The Figure shows the bleeding curve during 30-day follow-up period in patients treated with clopidogrel (blue line), prasugrel (red line) or ticagrelor (green line)

Unadjusted moderate and severe bleeding events of surviving patients during 1-year follow-up were not significantly different and numerically highest in clopidogrel (16.1% [36/223]) vs. prasugrel (12.3% [14/114]) vs. ticagrelor (6.7% [6/89])-treated patients, p = 0.081). Moderate bleeding events during 1-year follow-up were significantly different and highest in clopidogrel (15.2% [34/223]) compared to prasugrel (7.9% [9/114]) and ticagrelor (6.7% [6/89], *p* = 0.039) treated patients. The rate of in-hospital bleeding events was significantly higher in clopidogrel vs. prasugrel and vs. ticagrelor treated patients (18.5% [94/507] vs. 14.1% [25/177] vs. 9.9% [17/171], *p* = 0.022), Figure 3. This effect was mainly driven by a difference in moderate in-hospital bleedings in clopidogrel vs. prasugrel and vs. ticagrelor treated patients (16.4% [83/507] vs. 9.0% [16/177] vs. 6.4% [11/171], *p* < 0.001). In a multivariate Cox regression analysis of in-hospital moderate or severe bleeding events in surviving patients, ticagrelor was associated with a significantly lower risk compared to clopidogrel (Odds ratio [OR]: 0.*46*, 95% CI 0.23–0.90, *p* = 0*.*024) (Tables 4, 5, Supplemental Table 7). There was no sig*nificant* difference between ticagrelor- and prasugrel-treated patients (OR: 0.58, 95% CI 0.27–1.23, *p* = 0.16). Likewise, no significant difference was seen in the prasugrel vs. the clopidogrel group after adjustment (OR: 0.79, 95% CI 0.46–1.35,* p* = 0.39). Concerning moderate or severe bleeding events in survivors during 1-year follow-up, ticagrelor was associated with a significantly lower risk compared to both clopidogrel (HR: 0.43, 95% CI 0.25–0.72, *p* = 0.002) and prasugrel (HR: 0.51, 95% CI 0.28–0.95, *p* = 0.033). There was no significant difference in the 1-year bleeding risk between prasugrel- vs. clopidogrel-treated patients (HR 0.83 (CI 0.54–1.28, *p* = 0.40).

## Discussion

The key findings of our pooled sub-analysis of the randomized trials IABP-SHOCK II and CULPRIT-SHOCK are: (1) The frequency of the three different oral administered ADP-receptor antagonists changed during the study periods of both trials as clopidogrel was the predominant ADP-receptor antagonist in IABP-SHOCK II and ticagrelor in CULPRIT-SHOCK; (2) there was no statistically significant difference in the adjusted mortality risk in prasugrel vs. clopidogrel and in ticagrelor vs. clopidogrel treated patients; (3) the adjusted rate of in-hospital and 1-year bleedings was significantly lower in ticagrelor vs. clopidogrel treated patients.

This work represents the largest analysis of the safety and efficacy of the different orally available ADP-receptor antagonists clopidogrel, prasugrel and ticagrelor in patients with AMI complicated by cardiogenic shock. The landmark trials comparing clopidogrel vs. prasugrel [6], or vs. ticagrelor, respectively [7], excluded patients with cardiogenic shock prior to randomization. Just recently, the ISAR-REACT-5 trial demonstrated the superiority of a no pre-treatment strategy with prasugrel loading after coronary angiography over a pre-treatment strategy with ticagrelor in patients with AMI concerning 1-year adverse cardiovascular events. Whether the strategy or the drug investigated in this trial was superior cannot be concluded without a 2 × *2 factorial design.* Moreover, only 1.6% of the study participants suffered from cardiogenic shock [12]. Hence, there was a lack of evidence on the safety and efficacy of oral ADP-receptor antagonists in cardiogenic shock patients. Data are mainly limited to a previous sub-analysis of the IABP-SHOCK II trial showing that the newer and more potent prasugrel can be safely administered without harm in these patients. However, ticagrelor was barely investigated since only 3.4% of patients (*n *= 17) were treated with ticagrelor in IABP-SHOCK II. Since ticagrelor has become the predominantly administered ADP-receptor inhibitor in CULPRIT-SHOCK, we could now investigate safety and efficacy of this direct acting drug in this combined patient cohort including 170 patients on ticagrelor treatment (Supplemental Table 1). Of note, the use of ticagrelor—instead of prasugrel which is not recommended in patients above 75 years of age and below 60 kg of body weight —is not restricted and patient characteristics were more similar to the clopidogrel group, which is in large contrast to the prasugrel group in our investigation. However, adjusted mortality rates of all 3 cohorts in our sub-analysis were comparable and, therefore, correspond to the results of the IABP-SHOCK II sub-analysis [8].

Bleeding complications in general occur frequently in patients with AMI complicated by cardiogenic shock, and they are associated with mortality and are reported as high as in our study [13]. Cardiogenic shock patients are predisposed to suffer specifically from ICU-bleeding events due to their temporary need for resuscitation, requiring repeated chest compression and frequent puncture of both arteries and central veins. Temporary mechanical circulatory support devices, such as veno-arterial extracorporeal membrane oxygenation (ECMO) or co-axial pumps requiring large bore vascular access and therapeutic anticoagulation have also emerged as pivotal risk factors for bleeding events [14, 15]. By including patients from two large randomized controlled trials in our sub-analysis, ticagrelor was shown to be an independent predictor of lower bleeding risk compared to clopidogrel. Still, this finding might be explained by higher comorbidity of the clopidogrel group which cannot be completely eliminated by statistical adjustment. However, although ticagrelor was associated with less bleeding events compared to clopidogrel this did not translate into a reduced mortality—a finding which requires investigation in future trials.

### Pharmacological effects of ADP-receptor antagonists in cardiogenic shock and their role in bleeding events

There are some known pharmacodynamic disparities with respect to the antiplatelet action of clopidogrel, prasugrel and ticagrelor in AMI patients suffering from cardiogenic shock. The available data are limited to smaller and predominantly retrospective cohorts [16]. A reduced enteral absorption is a key drawback of oral ADP-receptor antagonists in cardiogenic shock [17]. Therefore, cangrelor, an intravenous ADP-receptor antagonist requiring no bioactivation *in-vivo*, might pose a suitable alternative [17] and is currently under investigation in the ongoing DAPT-SHOCK-AMI (NCT03551964) trial. Cangrelor did not have FDA-approval during the IABP-SHOCK-II trial and was barely used in the CULPRIT-SHOCK trial. Thus, we excluded this drug from our current analysis. Still, clopidogrel is known to be associated with a relatively slow onset of action and to induce lower levels of platelet inhibition in patients with AMI complicated by cardiogenic shock [18]. Consequently, current guidelines recommend to use prasugrel or ticagrelor in these patients. As our data show, the choice of the ADP-receptor antagonist on the other hand does not seem to be decisive for the bleeding risk in patients with AMI complicated by cardiogenic shock. Maybe, not the choice of the ADP-receptor antagonist itself might drive bleeding events, but rather the individual effect of the drug on platelet aggregation as reflected by differences in the quantifiable platelet reactivity. Still, this would not explain the differences in bleeding complications observed in this study since it is plausible that ticagrelor would have achieved a higher level of platelet inhibition than clopidogrel. An investigation, whether switching patients with low platelet reactivity on potent ADP-receptor antagonist to the weaker clopidogrel leads to fewer bleeding events in cardiogenic shock patients, appears to be very interesting—a strategy which was already shown to be safe in AMI patients without cardiogenic shock in the Testing Responsiveness to Platelet Inhibition on Chronic Antiplatelet Treatment For Acute Coronary Syndromes (TROPICAL-ACS) trial [19].

## Limitations

This study is a non-randomized pooled post-hoc sub-analysis with all its known limitations. Given the limited number of patients and the major differences in baseline and procedural characteristics arising from a pooled analysis of two heterogeneous trials, the possibility of type 2 errors cannot be completely excluded. Although the differences in patient characteristics highlight a treatment bias between the three subgroups, a risk adjusted analysis was used to take these limitations into account. No information was available on the prescription of ADP- receptor antagonists after discharge. 430 patients had to be excluded from this analysis according to exclusion criteria. Moreover, there was no platelet function monitoring of patients so that we could not analyse the pharmacodynamics of the different ADP-receptor antagonists. Likewise, the effects of shock on enteral absorption, as wells as an impaired activation of prodrugs (clopidogrel, prasugrel) were not addressed. No data were available to reflect the true renal function at baseline and during follow-up since the eGFR can only be calculated in a steady state. Although we could not analyse bleeding complications according to the latest BARC criteria, the classification according to GUSTO criteria was available in both trials.

## Summary

To date, this study represents the largest analysis with respect to the application of different ADP-receptor antagonists in patients with AMI complicated by cardiogenic shock. There were no significant differences in terms of mortality rates between patients treated with clopidogrel, prasugrel and ticagrelor after adjustment for differences in baseline characteristics. In addition, ticagrelor appears to be associated with a lower rate of in-hospital bleeding events compared to clopidogrel. However, the results must be interpreted with caution due to the limitations inherit to non-randomized pooled post-hoc sub-analyses. Contemporary studies in stable patients connect bleeding events rather to the pharmacological effect in the individual patient as opposed to a specific ADP-receptor antagonist. Thus, future trials may test whether a platelet reactivity guided choice of the ADP- receptor antagonist might be beneficial in terms of reducing bleeding events in patients with cardiogenic shock.

## Supplementary Information

Below is the link to the electronic supplementary material.

Supplementary file1 (DOCX 132 KB)

## Data Availability

All available data are incorporated into the manuscript and its online supplementary material.

## Abbreviations

AMI: Acute myocardial infarction
BARC: Bleeding academic research consortium
BMS: Bare-metal stent
CABG: Coronary artery bypass graft
CI: Confidence interval
DES: Drug-eluting stent
ECMO: Extracorporeal membrane oxygenation
HR: Hazard ratio
IABP: Intraaortic balloon pump
IQR: Interquartile range
LVEF: Left ventricular ejection fraction
OR: Odds ratio
PCI: Percutaneous coronary intervention
SAPS: Simplified acute physiology score
STEMI: ST-elevation myocardial infarction
RCT: Randomized controlled trial

## References

[1] Backhaus T, Fach A, Schmucker J, Fiehn E, Garstka D (2018). Stehmeier J Management and predictors of outcome in unselected patients with cardiogenic shock complicating acute ST-segment elevation myocardial infarction: results from the Bremen STEMI Registry. Clin Res Cardiol.

[2] Rathod KS, Koganti S, Iqbal MB, Jain AK, Kalra SS, Astroulakis Z (2017). Contemporary trends in cardiogenic shock: Incidence, intra-aortic balloon pump utilisation and outcomes from the London Heart Attack Group. Eur Heart J Acute Cardiovasc Care.

[3] Thiele H, Zeymer U, Neumann F-J, Ferenc M, Olbrich H-G, Hausleiter J (2012). Intraaortic balloon support for myocardial infarction with cardiogenic shock. N Engl J Med.

[4] Scherer C, Kupka D, Stocker TJ, Joskowiak D, Scheuplein H, Schönegger CM (2020). Isoflurane sedation in patients undergoing venoarterial extracorporeal membrane oxygenation treatment for cardiogenic shock-an observational propensity-matched study. Crit Care Explor March.

[5] Ibanez B, James S, Agewall S, Antunes MJ, Bucciarelli-Ducci C, Bueno H (2017). ESC Guidelines for the management of acute myocardial infarction in patients presenting with ST-segment elevation: The Task Force for the management of acute myocardial infarction in patients presenting with ST-segment elevation of the European Society of Cardiology (ESC). Eur Heart J.

[6] Wiviott SD, Braunwald E, McCabe CH, Montalescot G, Ruzyllo W, Gottlieb S (2007). Prasugrel versus clopidogrel in patients with acute coronary syndromes. N Engl J Med.

[7] Wallentin L, Becker RC, Budaj A, Cannon CP, Emanuelsson H, Held C (2009). Ticagrelor versus clopidogrel in patients with acute coronary syndromes. N Engl J Med.

[8] Orban M, Limbourg T, Neumann F-J, Ferenc M, Olbrich H-G, Richardt G (2016). ADP receptor antagonists in patients with acute myocardial infarction complicated by cardiogenic shock: a post hoc IABP-SHOCK II trial subgroup analysis. EuroIntervention.

[9] Orban M, Mayer K, Morath T, Bernlochner I, Hadamitzky M, Braun S (2014). Prasugrel vs clopidogrel in cardiogenic shock patients undergoing primary PCI for acute myocardial infarction. Thromb Haemost.

[10] Thiele H, Akin I, Sandri M, Fuernau G, de Waha S, Meyer-Saraei R (2017). PCI Strategies in Patients with Acute Myocardial Infarction and Cardiogenic Shock. N Engl J Med.

[11] Sabatine MS, Morrow DA, Giugliano RP, Burton Paul BJ, Murphy SA, McCabe CH (2005). Association of hemoglobin levels with clinical outcomes in acute coronary syndromes. Circulation.

[12] Schüpke S, Neumann F-J, Menichelli M, Mayer K, Bernlochner I, Wöhrle J (2019). Ticagrelor or Prasugrel in Patients with Acute Coronary Syndromes. N Engl J Med.

[13] Benedikt S, Karim I, Tobias L, Nikos W, Jan-Malte S, Federico P (2019). Impella support for acute myocardial infarction complicated by cardiogenic shock. Circulation.

[14] Freund Anne, Jobs Alexander, Lurz Philipp, Feistritzer Hans-Josef, de Waha-Thiele Suzanne, Meyer-Saraei Roza (2020) Frequency and impact of bleeding on outcome in patients with cardiogenic shock. JACC Cardiovasc Interv 13(10):1182–1193 10.1016/j.jcin.2020.02.042 32438988

[15] Scherer C, Lüsebrink E, Kupka D, Stocker TJ, Stark K, Stremmel C (2020). Long-term clinical outcome of cardiogenic shock patients undergoing impella cp treatment vs standard of care. J Clin Med.

[16] Elbadawi A, Elgendy IY, Mohamed AH, Barssoum K, Alotaki E, Ogunbayo GO (2018). Clopidogrel Versus Newer P2Y12 antagonists for percutaneous coronary intervention in patients with out-of-hospital cardiac arrest managed with therapeutic hypothermia: a meta-analysis. Cardiol Ther.

[17] Grimfjärd P, Lagerqvist B, Erlinge D, Varenhorst C, James S (2019). Clinical use of cangrelor: nationwide experience from the Swedish Coronary Angiography and Angioplasty Registry (SCAAR). Eur Heart J Cardiovasc Pharmacother.

[18] Orban M, Mayer K, Morath T, Bernlochner I, Hadamitzky M, Braun S (2015). The impact of therapeutic hypothermia on on-treatment platelet reactivity and clinical outcome in cardiogenic shock patients undergoing primary PCI for acute myocardial infarction: Results from the ISAR-SHOCK registry. Thromb Res.

[19] Sibbing D, Aradi D, Jacobshagen C, Gross L, Trenk D, Geisler T (2017). Guided de-escalation of antiplatelet treatment in patients with acute coronary syndrome undergoing percutaneous coronary intervention (TROPICAL-ACS): a randomised, open-label, multicentre trial. The Lancet.

