# Supplementary material for: Clopidogrel vs. prasugrel vs. ticagrelor in patients with acute myocardial infarction complicated by cardiogenic shock: a pooled IABP-SHOCK II and CULPRIT-SHOCK trial sub-analysis
Source: Clin Res Cardiol. 2021 May 17;110(9):1493–503. doi: 10.1007/s00392-021-01866-3 (PMC8405498; doi:10.1007/s00392-021-01866-3)
Supplement: Supplementary file 1 — Supplementary file1 (DOCX 132 KB) [file 392_2021_1866_MOESM1_ESM.docx]

**Detailed description of the statistical analysis**

Categorical variables are presented as counts and percentages and were compared by chi-square test. Continuous variables are expressed as median and inter-quartile-range (IQR) or mean and standard deviation and were compared using the Kruskal-Wallis test. The impact of acute medication on mortality and bleeding was examined in unadjusted and adjusted regression analyses and corresponding odds ratios (OR) or hazard ratios (HR) with 95%-confidence intervals (CI) are presented. *We included variables in both models that show an association with at least one of the two outcome variables (in-hospital bleeding or 1-year bleeding) by univariate analysis at p < 0.05*. For the bleeding model, we used a logistic regression for the in-hospital events and a Cox proportional hazards regression for the bleeding complications until the end of follow-up. *The timing of a bleeding event is not included in the endpoint “intrahospital bleeding”, therefore we chose logistic regression for analysing this endpoint. Regarding 1-year bleeding events the timing of the event, as well as the few patients lost-to-follow-up, have to be included in the analysis. Since logistic regression does not include these patients, we applied a Cox regression model for this endpoint. Our Cox regression model includes both patients with an early bleeding event and patients who were lost-to-follow-up.* The following variables were entered *in both multivariable models*: age and acute medication as fixed parameter, gender, previous myocardial infarction, resuscitation within 24h before randomization, mechanical ventilation, creatinine on admission [µmol/l], lactate > 2mmol/l on admission, treatment with unfractionated heparin and active mechanical circulatory support. The models were finalized with a forward selection using an entry level of p<0.05. Concerning the model for mortality, variables entered in the model were age, female, previous myocardial infarction, previous PCI, previous coronary artery bypass graft (CABG) surgery, previous stroke, known renal insufficiency (glomerular filtration rate, GFR < 30 ml/min), resuscitation within 24h before randomization, ST-segment elevation, creatinine on admission [µmol/l], heart rate [bpm] before PCI, systolic blood pressure [mmHg] before PCI, SAPS II Score. The cumulative incidence of mortality and bleeding event rates were estimated using the Product-Limit method and were visualized as Kaplan-Meier curves. Non-surviving patients and patients without event were censored. All p-values were two-tailed and <0.05 was considered statistically significant. All analyses were performed using SAS statistical package version 9.4 (Cary, North Carolina, USA).

**Supplemental Tables**

**Supplemental Table 1. Distribution of the different ADP-receptor inhibitors in the respective randomised trials**

|  |  |  |  |
| --- | --- | --- | --- |
| **Therapeutic agent** | **IABP-SHOCK II** | **CULPRIT-SHOCK** | **Pooled** |
|  |  |  |  |
|  |  |  |  |
| **Clopidogrel** | 387 (77.9) | 120 (33.4) | 507 (59.2) |
|  |  |  |  |
| **Prasugrel** | 93(18.7) | 85 (23.7) | 178 (20.8) |
|  |  |  |  |
| **Ticagrelor** | 17 (3.4) | 154 (42.9) | 171 (20.0) |
|  |  |  |  |
| **Σ** | 497 (58.0) | 359 (42.0) | 856 |
|  |  |  |  |
|  | **Value** | **df** |  |
| **Pearson Chi-Square** | 234.6 | 2 |  |

**Legend to Supplemental Table 1:**

This table shows the distribution of the different ADP-receptor inhibitors used in both the IABP-SHOCK II and CULPRIT-SHOCK trial. Values are depicted as number of patients (percentages).

**Supplemental Table 2. Detailed baseline characteristics of the study cohorts**

|  |  |  |  |  |
| --- | --- | --- | --- | --- |
| **Variable** | **Clopidogrel** | **Prasugrel** | **Ticagrelor** | **P value** |
|  | **n=507** | **n=178** | **n=171** |  |
|  |  |  |  |  |
|  |  |  |  |  |
| **Age, years ± SD** | 69 ± 12 | 62 ± 11 | 69 ± 12 | **<0.001** |
|  |  |  |  |  |
| **Female gender, n (%)** | 160 (31.6) | 41(23.0) | 42 (24.6) | **0.044** |
|  |  |  |  |  |
| **Body-mass index*, median (IQR)** | 27.2 (24.5, 30.1) | 26.3 (24.2, 28.9) | 27.7 (24.7, 29.4) | 0.77 |
|  |  |  |  |  |
| **Cardiovascular risk factors – no./total no. (%)** |  |  |  |  |
|  |  |  |  |  |
| **Current smoking** | 144/501 (28.7) | 80/176 (45.5) | 46/167 (27.5) | **< 0.001** |
|  |  |  |  |  |
| **Hypertension** | 353/504 (70) | 112/178 (62.9) | 92/170 (54.1) | **< 0.001** |
|  |  |  |  |  |
| **Hypercholesterolemia** | 187/503 (37.2) | 67/178 (37.6) | 55/169 (32.5) | 0.51 |
|  |  |  |  |  |
| **Diabetes mellitus** | 180/505 (35.6) | 35/178 (19.7) | 51/169 (30.2) | **< 0.001** |
|  |  |  |  |  |
| **Vital signs at admission, median (IQR)** |  |  |  |  |
| **Heart rate [beats per minute]** | 90 (75, 110) | 90 (69, 110) | 80 (66, 100) | **0.002** |
|  |  |  |  |  |
| **Systolic blood pressure [mmHg]** | 90 (80, 110) | 100 (81, 117) | 98 (80, 122) | **0.007** |
|  |  |  |  |  |
| **Diastolic blood pressure [mmHg]** | 59 (49, 70) | 60 (50, 77) | 60 (49, 80) | **0.004** |
|  |  |  |  |  |
| **Morbidities** |  |  |  |  |
|  |  |  |  |  |
| **Prior myocardial infarction, no./total no. (%)** | 121/506 (23.9) | 35/178 (19.7) | 19/170 (11.2) | **0.002** |
|  |  |  |  |  |
| **Prior stroke, no./total no. (%)** | 51/506 (10.1) | 3/178 (1.7) | 9/169 (5.3) | **< 0.001** |
|  |  |  |  |  |
| **Peripheral arterial disease, no./total no. (%)** | 72/506 (14.2) | 18/178 (10.1) | 13/170 (7.6) | 0.050 |
|  |  |  |  |  |
| **Prior PCI, no./total no. (%)** | 108/506 (21.3) | 39/178 (21.9) | 22/170 (12.9) | **0.043** |
|  |  |  |  |  |
| **Prior CABG surgery, no./total no. (%)** | 28/506 (5.5) | 2/178 (1.1) | 4/170 (2.4) | **0.017** |
|  |  |  |  |  |
| **Renal impairment (GFR <30 ml/min), no./total no. (%)** | 106/506 (20.9) | 13/178 (7.3) | 13/169 (7.7) | **< 0.001** |
|  |  |  |  |  |
| **Chronic drug therapy, no./total no. (%)** |  |  |  |  |
|  |  |  |  |  |
| **ASA** | 210/473 (44.4) | 50/163 (30.7) | 51/140 (36.4) | **0.005** |
|  |  |  |  |  |
| **Clopidogrel** | 72/474 (15.2) | 10/162 (6.2) | 6/135 (4.4) | **< 0.001** |
|  |  |  |  |  |
| **Prasugrel** | 1/474 (0.2) | 3/162 (1.9) | 0/135 (0.0) | **0.028** |
|  |  |  |  |  |
| **Ticagrelor** | 2/358 (0.6) | 1/137 (0.7) | 15/136 (11.0) | **< 0.001** |
|  |  |  |  |  |
| **Vitamin K-antagonists** | 31/473 (6.6) | 4/161 (2.5) | 3/134 (2.2) | **0.034** |
|  |  |  |  |  |
| **Beta-blocker** | 210/471 (44.6) | 50/160 (31.3) | 38/135 (28.1) | **< 0.001** |
|  |  |  |  |  |
| **ACE-inhibitors/ARB** | 222/471 (47.1) | 56/160 (35.0) | 55/137 (40.1) | **0.020** |
|  |  |  |  |  |
| **Statins** | 150/472 (31.8) | 41/160 (25.6) | 36/135 (26.7) | 0.24 |
|  |  |  |  |  |
|  |  |  |  |  |

**Legend to Supplemental Table 2:**

This table shows the detailed baseline characteristics of the clopidogrel, the prasugrel and the ticagrelor subgroup. Data presented are means (± standard deviation, SD), medians [interquartile range, IQR] or numbers of patients (percentages). *The body-mass index is the weight in kilograms divided by the square of the height in meters. PCI, percutaneous coronary intervention; CABG, coronary artery bypass graft; GFR, glomerular filtration rate; ASA, acetylsalicylic acid; ACE, angiotensin converting enzyme; ARB, angiotensin receptor blockers. P-values: Pearson chi-squared test or Mann-Whitney-Wilcoxon test.

**Supplemental Table 3. Detailed clinical presentation, laboratory parameters and ECG of all subgroups prior to randomization during the primary trials**

|  |  |  |  |  |
| --- | --- | --- | --- | --- |
| **Variable** | **Clopidogrel** | **Prasugrel** | **Ticagrelor** | **P value** |
|  | **n=507** | **n=178** | **n=171** |  |
|  |  |  |  |  |
|  |  |  |  |  |
| **Signs of impaired organ perfusion, no./total no. (%)** |  |  |  |  |
|  |  |  |  |  |
| **Altered mental status** | 362/506 (71.5) | 126/178 (70.8) | 116/170 (68.2) | 0.71 |
|  |  |  |  |  |
| **Cold, clammy skin and extremities** | 420/506 (83.0) | 143/178 (80.3) | 111/168 (66.1) | **< 0.001** |
|  |  |  |  |  |
| **Oliguria (≤20 ml/h)** | 151/506 (29.8) | 50/176 (28.4) | 36/166 (21.7) | 0.13 |
|  |  |  |  |  |
| **pH <7.36** | 316/505 (62.6) | 90/174 (51.7) | 106/167 (63.5) | **0.028** |
|  |  |  |  |  |
| **Serum lactate >2.0 mmol/litre** | 373/505 (73.9) | 112/174 (64.4) | 108/168 (64.3) | **0.012** |
|  |  |  |  |  |
| **Resuscitation, no./total no. (%)** | 204/506 (40.3) | 87/178 (48.9) | 85/171 (49.7) | **0.034** |
|  |  |  |  |  |
| **Mechanical ventilation, no./total no. (%)** | 275/506 (54.3) | 90/178 (50.6) | 97/171 (56.7) | 0.50 |
|  |  |  |  |  |
| **Acute LVEF, median (IQR)** | 35 (25, 44) | 39 (30, 45) | 35 (25, 40) | 0.13 |
|  |  |  |  |  |
| **SAPS II, median (IQR)** | 52 (36, 69) | 44 (28.5, 60) | 49.5 (31.5, 74.5) | **< 0.001** |
|  |  |  |  |  |
| **Body temperature (°C), median (IQR)** | 36.6 (35.4, 37.3) | 36.4 (35.0, 37.2) | 36.2 (35.1, 37.0) | 0.087 |
|  |  |  |  |  |
| **ECG prior to PCI, no./total no. (%)** |  |  |  |  |
|  |  |  |  |  |
| **Pacemaker rhythm** | 12/506 (2.4) | 6/177 (3.4) | 4/168 (2.4) | 0.75 |
|  |  |  |  |  |
| **Left bundle branch block** | 64/506 (12.6) | 18/176 (10.2) | 28/167 (16.8) | 0.19 |
|  |  |  |  |  |
| **ST-segment elevation** | 296/506 (58.5) | 143/176 (81.3) | 118/167 (70.7) | **< 0.001** |
|  |  |  |  |  |
| **Anterior infarction** | 192/504 (38.1) | 80/175 (45.7) | 71/166 (42.8) | 0.17 |
|  |  |  |  |  |
| **Non-anterior infarction** | 149/504 (29.6) | 63/175 (36.0) | 46/166 (27.7) | 0.19 |
|  |  |  |  |  |
| **Other rhythm** | 44/507 (8.7) | 26/177 (14.7) | 23/169 (13.6) | **0.039** |
|  |  |  |  |  |
|  |  |  |  |  |
| **Laboratory parameters, median (IQR)** |  |  |  |  |
|  |  |  |  |  |
| **Glucose (mmol/l)** | 11.63 (8.00, 16.46) | 10.40 (8.10, 15.43) | 11.83 (8.40, 17.26) | 0.25 |
|  |  |  |  |  |
| **Creatinine (µmol/l)** | 116.50 (96.00, 151.00) | 102.27 (86.32, 133.30) | 108.86 (91.05, 136.00) | **< 0.001** |
|  |  |  |  |  |
| **Haemoglobin (mmol/l)** | 8.10 (7.01, 9.10) | 8.75 (7.94, 9.31) | 8.40 (7.57, 9.06) | **< 0.001** |
|  |  |  |  |  |
| **GFR (Cockcroft/Gault formula [ml/min])** | 49.89 (34.53, 67.04) | 70.39 (56.29, 94.81) | 62.00 (45.46, 84.60) | **< 0.001** |
|  |  |  |  |  |
|  |  |  |  |  |

**Legend to Supplemental Table 3:**

This table shows the detailed clinical presentation and laboratory parameters of all three cohorts; Data presented are means (± standard deviation, SD), medians (interquartile range, IQR) or numbers of patients (percentages). LVEF, left ventricular ejection fraction; SAPS II, simplified acute physiology score II; ECG, electrocardiogram; PCI, percutaneous coronary intervention; GFR, glomerular filtration rate. P-values: Pearson chi-squared test or Mann-Whitney-Wilcoxon test.

**Supplemental Table 4. Detailed acute drug therapy and interventions of the study groups**

|  |  |  |  |  |
| --- | --- | --- | --- | --- |
| **Variable** | **Clopidogrel** | **Prasugrel** | **Ticagrelor** | **P value** |
|  | **n=507** | **n=178** | **n=171** |  |
|  |  |  |  |  |
|  |  |  |  |  |
| **No. of diseased vessels, no./total no. (%)** |  |  |  |  |
|  |  |  |  |  |
| **1** | 80/503 (15.9) | 25/178 (14.0) | 3/171 (1.8) |  |
|  |  |  |  |  |
| **2** | 145/503 (28.8) | 62/178 (34.8) | 64/171 (37.4) |  |
|  |  |  |  |  |
| **3** | 278/503 (55.3) | 91/178 (51.1) | 104/171 (60.8) |  |
|  |  |  |  |  |
| **Artery with culprit lesion, no./total no. (%)** |  |  |  |  |
|  |  |  |  |  |
| **Right coronary artery** | 131/503 (26.0) | 60/178 (33.7) | 37/171 (21.6) |  |
|  |  |  |  |  |
| **Left main** | 42/503 (8.3) | 12/178 (6.7) | 15/171 (8.8) |  |
|  |  |  |  |  |
| **Left anterior descending** | 211/503 (41.9) | 72/178 (40.4) | 83/171 (48.5) |  |
|  |  |  |  |  |
| **Circumflex branch** | 106/503 (21.1) | 33/178 (18.5) | 36/171 (21.1) |  |
|  |  |  |  |  |
| **Bypass** | 11/502 (2.2) | 1/178 (0.6) | 0/171 (0.0) |  |
|  |  |  |  |  |
| **Stent implanted in lesions, no./total no. (%)** | 466/496 (94.0) | 171/177 (96.6) | 162/170 (95.3) | 0.37 |
|  |  |  |  |  |
| **Bare-metal stent culprit lesion** | 234/466 (50.2) | 41/171 (24.0) | 13/162 (8.0) | **< 0.001** |
|  |  |  |  |  |
| **Drug-eluting stent culprit lesion** | 245/466 (52.6) | 130/171 (76.0) | 149/162 (92.0) | **< 0.001** |
|  |  |  |  |  |
| **Bioabsorbable scaffold culprit lesion** | 0/111 (0.0) | 0/83 (0.0) | 2/147 (1.4) | 0.27 |
|  |  |  |  |  |
| **Manual thrombectomy before stenting, no./total no. (%)** | 90/506 (17.8) | 60/178 (33.7) | 40/171 (23.4) | **< 0.001** |
|  |  |  |  |  |
| **Manual thrombectomy after stenting, no./total no. (%)** | 15/506 (3.0) | 2/178 (1.1) | 3/171 (1.8) | 0.32 |
|  |  |  |  |  |
| **Stent thrombosis, no./total no. (%)** | 4/506 (0.8) | 5/178 (2.8) | 2/171 (1.2) | 0.12 |
|  |  |  |  |  |
| **Immediate PCI of additional lesions, no./total no. (%)** | 169/497 (34.0) | 63/177 (35.6) | 86/170 (50.6) | **< 0.001** |
|  |  |  |  |  |
| **Indication for immediate CABG, no./total no. (%)** | 7/447 (1.6) | 1/159 (0.6) | 1/168 (0.6) | 0.47 |
|  |  |  |  |  |
| **Mild induced Hypothermia, no./total no. (%)** | 146/507 (28.8) | 67/178 (37.6) | 51/170 (30.0) | 0.086 |
|  |  |  |  |  |
| **Catecholamine requirement, no./total no. (%)** | 459/507 (90.5) | 151/177 (85.3) | 151/171 (88.3) | 0.15 |
|  |  |  |  |  |
| **Epinephrine** | 166/459 (36.2) | 57/151 (37.7) | 67/151 (44.4) | 0.20 |
|  |  |  |  |  |
| **Norepinephrine** | 400/459 (87.1) | 133/151 (88.1) | 125/151 (82.8) | 0.32 |
|  |  |  |  |  |
| **Dobutamine** | 283/459 (61.7) | 96/151 (63.6) | 67/151 (44.4) | **< 0.001** |
|  |  |  |  |  |
| **Dopamine** | 59/459 (12.9) | 8/151 (5.3) | 5/151 (3.3) | **< 0.001** |
|  |  |  |  |  |
| **Mechanical ventilation, no./total no. (%)** | 407/507 (80.3) | 126/177 (71.2) | 126/171 (73.7) | **0.023** |
|  |  |  |  |  |
| **Invasive** | 391/407 (96.1) | 117/126 (92.9) | 112/126 (88.9) | **0.009** |
|  |  |  |  |  |
| **Non-invasive** | 55/407 (13.5) | 28/126 (22.2) | 23/126 (18.3) | 0.051 |
|  |  |  |  |  |
| **Acute drug therapy, no./total no. (%)** |  |  |  |  |
|  |  |  |  |  |
| **Aspirin** | 474/507 (93.5) | 163/178 (91.6) | 144/171 (84.2) | **0.001** |
|  |  |  |  |  |
| **Clopidogrel** | 507/507 (100.0) | 0/178 (0.0) | 0/171 (0.0) | **---** |
|  |  |  |  |  |
| **Prasugrel** | 0/507 (0.0) | 178/178 (100.0) | 0/171 (0.0) | **---** |
|  |  |  |  |  |
| **Ticagrelor** | 0/391 (0.0) | 0/156 (0.0) | 171/171 (100.0) | **---** |
|  |  |  |  |  |
| **GP IIb/IIIa-Inhibitors** | 189/507 (37.3) | 79/178 (44.4) | 44/171 (25.7) | **0.001** |
|  |  |  |  |  |
| **UF Heparin** | 467/507 (92.1) | 154/178 (86.5) | 143/171 (83.6) | **0.003** |
|  |  |  |  |  |
| **LMW Heparin** | 29/507 (5.7) | 21/178 (11.8) | 20/171 (11.7) | **0.007** |
|  |  |  |  |  |
| **Bivalirudin** | 27/507 (5.3) | 31/178 (17.4) | 9/171 (5.3) | **< 0.001** |
|  |  |  |  |  |
| **Drug therapy at discharge, no./total no. (%)** |  |  |  |  |
|  |  |  |  |  |
| **Aspirin** | 436/492 (88.6) | 159/173 (91.9) | 147/167 (88.0) | 0.42 |
|  |  |  |  |  |
| **Clopidogrel** | 399/492 (81.1) | 18/173 (10.4) | 26/167 (15.6) | **< 0.001** |
|  |  |  |  |  |
| **Prasugrel** | 41/491 (8.4) | 137/173 (79.2) | 8/167 (4.8) | **< 0.001** |
|  |  |  |  |  |
| **Ticagrelor** | 12/402 (3.0) | 7/157 (4.5) | 128/168 (76.2) | **< 0.001** |
|  |  |  |  |  |
| **Vitamin K-antagonists** | 34/490 (6.9) | 11/173 (6.4) | 8/167 (4.8) | 0.62 |
|  |  |  |  |  |
| **ACE-inhibitors/ARB** | 279/490 (56.9) | 121/173 (69.9) | 97/167 (58.1) | **0.010** |
|  |  |  |  |  |
| **Beta-blocker** | 292/489 (59.7) | 129/172 (75.0) | 99/167 (59.3) | **< 0.001** |
|  |  |  |  |  |
| **Diuretics** | 310/491 (63.1) | 105/173 (60.7) | 82/167 (49.1) | **0.006** |
|  |  |  |  |  |
| **Aldosterone-Antagonist** | 114/488 (23.4) | 38/173 (22.0) | 36/167 (21.6) | 0.86 |
|  |  |  |  |  |
| **Calcium-Antagonist** | 52/489 (10.6) | 20/173 (11.6) | 13/167 (7.8) | 0.47 |
|  |  |  |  |  |
| **Statins** | 356/492 (72.4) | 144/173 (83.2) | 110/167 (65.9) | **0.001** |
|  |  |  |  |  |
| **Duration of mechanical ventilation [days], median (IQR)** | 3.0 (1.0, 8.0) | 4.0 (1.0, 9.0) | 2.0 (1.0, 6.0) | 0.26 |
|  |  |  |  |  |
| **Duration of ICU treatment [days], median (IQR)** | 5.5 (3.0, 11.0) | 6.0 (3.0, 14.0) | 4.0 (2.0, 10.5) | **0.024** |
|  |  |  |  |  |
| **Duration of catecholamine treatment [days], median (IQR)** | 3.0 (1.0, 5.0) | 3.0 (1.0, 6.0) | 2.0 (1.0, 4.0) | **0.018** |
|  |  |  |  |  |
| **Days to hemodynamic stabilization, median (IQR)** | 3.0 (1.0, 6.0) | 3.0 (1.0, 6.0) | 3.0 (1.0, 6.0) | 0.18 |
|  |  |  |  |  |
| **In-hospital bleeding events** | 94/507 (18.5) | 25/177 (14.1) | 17/171 (9.9) | **0.022** |
|  |  |  |  |  |
| **Severe/life-threatening** | 18/507 (3.6) | 12/177 (6.8) | 7/171 (4.1) | 0.19 |
|  |  |  |  |  |
| **Moderate** | 83/507 (16.4) | 16/177 (9.0) | 11/171 (6.4) | **< 0.001** |
|  |  |  |  |  |
|  |  |  |  |  |

**Legend to Supplemental Table 4:**

This table shows both the detailed acute drug therapy and interventions of all three cohorts, as well as unadjusted in-hospital bleeding events; Data presented are means (± standard deviation, SD), medians (inter quartile range, IQR) or numbers of patients (percentages). PCI, percutaneous coronary intervention; CABG, coronary artery bypass graft, GP IIb/IIIa, glycoprotein IIb/IIIa; UF, unfractionated; LMW, low molecular weight; ACE, angiotensin converting enzyme; ARB, angiotensin receptor blockers; ICU, intensive care unit. P-values: Pearson chi-squared test or Mann-Whitney-Wilcoxon test.

**Supplemental Table 5. *Logistic* regression model of in- hospital bleeding events**

|  |  |  |  |  |
| --- | --- | --- | --- | --- |
| **Variable** | **Moderate and severe bleedings** | **No bleeding** | **P value** | **OR (95% CI)** |
|  | **n= 215 (16.8%)** | **n= 1068 (83.2%)** |  |  |
|  |  |  |  |  |
|  |  |  |  |  |
| **Demographics, mean ± SD** |  |  |  |  |
|  |  |  |  |  |
| **Age [years]** | 68 ± 12 | 68 ± 12 | 0.71 |  |
|  |  |  |  |  |
| **Female gender, % (no./total no.)** | 34.9 % (75/215) | 25.6 % (273/1068) | **0.005** | **1.56 (1.14-2.13)** |
|  |  |  |  |  |
| **Weight [kg]** | 82 ± 15 | 82 ± 15 | 0.89 |  |
|  |  |  |  |  |
| **Height [cm]** | 172 ± 8 | 173 ± 9 | 0.25 |  |
|  |  |  |  |  |
| **BMI [kg/m²]** | 27.7 ± 4.7 | 27.7 ± 8.7 | 0.53 |  |
|  |  |  |  |  |
| **Cardiovascular risk factors, % (no./total no.)** |  |  |  |  |
|  |  |  |  |  |
| **Current smoking** | 30.0 % (63/210) | 30.3 % (315/1040) | 0.93 | 0.99 (0.71-1.36) |
|  |  |  |  |  |
| **Hypertension** | 60.9 % (131/215) | 65.3 % (686/1051) | 0.23 | 0.83 (0.61-1.12) |
|  |  |  |  |  |
| **Dyslipidemia** | 38.8 % (83/214) | 35.4 % (371/1048) | 0.35 | 1.16 (0.85-1.56) |
|  |  |  |  |  |
| **Diabetes mellitus** | 34.3 % (73/213) | 32.3 % (340/1052) | 0.58 | 1.09 (0.80-1.49) |
|  |  |  |  |  |
| **Vital signs at admission, mean ± SD** |  |  |  |  |
|  |  |  |  |  |
| **Heart rate [bpm]** | 93 ± 27 | 91 ± 28 | 0.11 |  |
|  |  |  |  |  |
| **Systolic blood pressure [mmHg]** | 97 ± 24 | 101 ± 29 | 0.12 |  |
|  |  |  |  |  |
| **Diastolic blood pressure [mmHg]** | 58 ± 17 | 63 ± 19 | 0.006 |  |
|  |  |  |  |  |
| **Medical history: Cardiovascular parameters, % (no./total no.)** |  |  |  |  |
|  |  |  |  |  |
| **Previous myocardial infarction** | 24.2 % (52/215) | 18.3 % (193/1055) | 0.046 | **1.42 (1.01-2.02)** |
|  |  |  |  |  |
| **Previous PCI** | 21.4 % (46/215) | 18.6 % (196/1054) | 0.34 | 1.19 (0.83-1.71) |
|  |  |  |  |  |
| **Previous CABG surgery** | 2.8 % (6/215) | 5.6 % (59/1059) | 0.091 | 0.49 (0.21-1.14) |
|  |  |  |  |  |
| **Previous stroke** | 7.9 % (17/215) | 7.2 % (76/1058) | 0.71 | 1.11 (0.64-1.92) |
|  |  |  |  |  |
| **Known peripheral artery disease** | 8.8 % (19/215) | 12.7 % (134/1059) | 0.12 | 0.67 (0.40-1.11) |
|  |  |  |  |  |
| **Known chronic kidney disease (eGFR < 30 ml/min)** | 18.1 % (39/215) | 12.4 % (131/1056) | **0.024** | **1.56 (1.06-2.32)** |
|  |  |  |  |  |
| **Chronic drug therapy, % (no./total no.)** |  |  |  |  |
|  |  |  |  |  |
| **Aspirin** | 42.7 % (82/192) | 39.7 % (365/920) | 0.44 | 1.13 (0.83-1.55) |
|  |  |  |  |  |
| **Clopidogrel** | 11.6 % (22/189) | 10.4 % (95/914) | 0.61 | 1.14 (0.69-1.86) |
|  |  |  |  |  |
| **Prasugrel** | 1.6 % (3/189) | 1.3 % (12/913) | 0.77 | 1.21 (0.34-4.33) |
|  |  |  |  |  |
| **Ticagrelor** | 1.9 % (3/158) | 2.4 % (19/782) | 0.69 | 0.78 (0.23-2.66) |
|  |  |  |  |  |
| **Vitamin-K-Antagonists** | 6.3 % (12/190) | 4.7 % (43/911) | 0.36 | 1.36 (0.70-2.63) |
|  |  |  |  |  |
| **Beta-Blocker** | 41.5 % (78/188) | 38.3 % (348/909) | 0.41 | 1.14 (0.83-1.57) |
|  |  |  |  |  |
| **ACE-inhibitors/ARB** | 42.6 % (80/188) | 44.8 % (408/910) | 0.57 | 0.91 (0.66-1.25) |
|  |  |  |  |  |
| **Statin** | 35.6 % (67/188) | 31.1 % (283/909) | 0.23 | 1.22 (0.88-1.70) |
|  |  |  |  |  |
| **Clinical and laboratory parameters, % (no./total no.)** |  |  |  |  |
|  |  |  |  |  |
| **Altered mental status** | 78.0 % (167/214) | 69.4 % (738/1064) | **0.011** | **1.57 (1.11-2.22)** |
|  |  |  |  |  |
| **Cold, clammy skin and extremities** | 79.7 % (169/212) | 75.7 % (800/1057) | 0.21 | 1.26 (0.88-1.82) |
|  |  |  |  |  |
| **Oliguria (≤ 30ml/h)** | 32.4 % (67/207) | 28.0 % (294/1049) | 0.21 | 1.23 (0.89-1.69) |
|  |  |  |  |  |
| **pH < 7.36** | 64.8 % (136/210) | 60.7 % (637/1049) | 0.27 | 1.19 (0.87-1.62) |
|  |  |  |  |  |
| **Serum-lactate > 2mmol/l** | 74.8 % (157/210) | 69.0 % (725/1050) | 0.099 | 1.33 (0.95-1.86) |
|  |  |  |  |  |
| **Mechanical ventilation** | 69.6 % (149/214) | 56.1 % (597/1065) | **< 0.001** | **1.80 (1.31-2.46)** |
|  |  |  |  |  |
| **Resuscitation within 24h before randomization** | 59.3 % (127/214) | 47.6 % (507/1066) | **0.002** | **1.61 (1.19-2.17)** |
|  |  |  |  |  |
| **ECG pre PCI, % (no./total no.)** |  |  |  |  |
|  |  |  |  |  |
| **Pacemaker rhythm** | 1.0 % (2/210) | 2.9 % (30/1050) | 0.11 | 0.33 (0.08-1.38) |
|  |  |  |  |  |
| **Left bundle branch block (LBBB)** | 15.2 % (32/210) | 13.5 % (142/1048) | 0.52 | 1.15 (0.76-1.74) |
|  |  |  |  |  |
| **ST-segment elevation** | 60.3 % (126/209) | 62.9 % (659/1048) | 0.48 | 0.90 (0.66-1.21) |
|  |  |  |  |  |
| **Anterior infarction** | 39.4 % (82/208) | 37.4 % (390/1044) | 0.57 | 1.09 (0.80-1.48) |
|  |  |  |  |  |
| **Non-anterior infarction** | 27.9 % (58/208) | 30.1 % (314/1044) | 0.53 | 0.90 (0.65-1.25) |
|  |  |  |  |  |
| **Other rhythm** | 11.4 % (24/210) | 12.5 % (132/1055) | 0.66 | 0.90 (0.57-1.43) |
|  |  |  |  |  |
| **Laboratory parameters at admission, mean ± SD** |  |  |  |  |
|  |  |  |  |  |
| **Glucose on Admission [mmol/l]** | 14.79 ± 12.65 | 16.01 ± 45.47 | 0.16 |  |
|  |  |  |  |  |
| **Creatinine on Admission [µmol/l]** | 136.79 ± 79.64 | 141.65 ± 390.55 | 0.10 |  |
|  |  |  |  |  |
| **Hemoglobin [mmol/l]** | 8.13 ± 6.76 | 9.50 ± 25.07 | < 0.001 |  |
|  |  |  |  |  |
| **eGFR (Cockcroft and Gault formula) [ml/min]** | 60.59 ± 28.56 | 81.00 ± 318.56 | 0.37 |  |
|  |  |  |  |  |
| **Body temperature** | 35.99 ± 1.55 | 36.19 ± 1.54 | 0.065 |  |
|  |  |  |  |  |
| **SAPS II** | 56.06 ± 24.15 | 51.31 ± 23.81 | 0.005 |  |
|  |  |  |  |  |
| **Acute LVEF [%], mean ± SD** | 36 ± 12 | 34 ± 13 | 0.21 |  |
|  |  |  |  |  |
| **Immediate PCI of additional lesions, % (no./total no.)** | 44.0 % (91/207) | 41.0 % (431/1051) | 0.43 | 1.13 (0.84-1.52) |
|  |  |  |  |  |
| **Indication for immediate CABG, % (no./total no.)** | 3.2 % (6/186) | 0.5 % (5/989) | < 0.001 | 6.56 (1.98-21.72) |
|  |  |  |  |  |
| **Mild induced Hypothermia, % (no./total no.)** | 40.5 % (87/215) | 31.7 % (338/1066) | 0.013 | 1.46 (1.08-1.98) |
|  |  |  |  |  |
| **Catecholamine requirement, % (no./total no.)** | 97.2 % (209/215) | 90.0 % (960/1067) | **< 0.001** | **3.88 (1.68-8.96)** |
|  |  |  |  |  |
| **Epinephrine** | 50.2 % (105/209) | 38.0 % (365/960) | **0.001** | **1.65 (1.22-2.22)** |
|  |  |  |  |  |
| **Norepinephrine** | 95.2 % (199/209) | 87.3 % (838/960) | **0.001** | **2.90 (1.49-5.62)** |
|  |  |  |  |  |
| **Dobutamine** | 61.7 % (129/209) | 58.2 % (559/960) | 0.35 | 1.16 (0.85-1.57) |
|  |  |  |  |  |
| **Dopamine** | 6.7 % (14/209) | 6.9 % (66/960) | 0.93 | 0.97 (0.54-1.77) |
|  |  |  |  |  |
| **Mechanical ventilation, % (no./total no.)** | 94.4 % (203/215) | 79.0 % (843/1067) | **< 0.001** | **4.50 (2.47-8.20)** |
|  |  |  |  |  |
| **Invasive** | 95.1 % (193/203) | 93.1 % (785/843) | 0.31 | 1.43 (0.72-2.84) |
|  |  |  |  |  |
| **Non-invasive** | 21.7 % (44/203) | 16.5 % (139/843) | 0.081 | 1.40 (0.96-2.05) |
|  |  |  |  |  |
| **Duration of all patients, median (IQR)** |  |  |  |  |
|  |  |  |  |  |
| **Duration of mechanical ventilation [day]** | 6.0 (3.0, 13.0) | 2.0 (1.0, 6.0) | < 0.001 |  |
|  |  |  |  |  |
| **Duration of ICU treatment [day]** | 10.0 (4.0, 18.0) | 5.0 (2.0, 11.0) | < 0.001 |  |
|  |  |  |  |  |
| **Duration of catecholamine therapy [day]** | 5.0 (2.0, 9.0) | 2.0 (1.0, 5.0) | < 0.001 |  |
|  |  |  |  |  |
| **Days to hemodynamic stabilization [day]** | 5.0 (3.0, 9.0) | 3.0 (1.0, 5.0) | < 0.001 |  |
|  |  |  |  |  |
| **Medication: Acute drug therapy, % (no./total no.)** |  |  |  |  |
|  |  |  |  |  |
| **Aspirin** | 84.7 % (182/215) | 80.8 % (861/1065) | 0.19 | 1.31 (0.88-1.95) |
|  |  |  |  |  |
| **Clopidogrel** | 45.1 % (97/215) | 40.4 % (430/1065) | 0.20 | 1.21 (0.90-1.63) |
|  |  |  |  |  |
| **Prasugrel** | 13.0 % (28/215) | 15.4 % (164/1065) | 0.37 | 0.82 (0.53-1.27) |
|  |  |  |  |  |
| **Ticagrelor** | 10.2 % (19/187) | 17.2 % (161/934) | 0.016 | 0.54 (0.33-0.90) |
|  |  |  |  |  |
| **GP IIb/IIIa-Inhibitors** | 37.7 % (81/215) | 32.6 % (347/1065) | 0.15 | 1.25 (0.92-1.70) |
|  |  |  |  |  |
| **UF Heparin** | 91.6 % (197/215) | 85.2 % (907/1065) | 0.012 | 1.91 (1.14-3.18) |
|  |  |  |  |  |
| **LMW Heparin** | 5.6 % (12/215) | 10.0 % (107/1065) | 0.040 | 0.53 (0.29-0.98) |
|  |  |  |  |  |
| **Bivalirudin** | 8.8 % (19/215) | 8.1 % (86/1065) | 0.71 | 1.10 (0.66-1.86) |
|  |  |  |  |  |
| **Medication at discharge, % (no./total no.)** |  |  |  |  |
|  |  |  |  |  |
| **Aspirin** | 81.9 % (172/210) | 84.9 % (880/1036) | 0.27 | 0.80 (0.54-1.19) |
|  |  |  |  |  |
| **Clopidogrel** | 49.0 % (103/210) | 45.6 % (472/1036) | 0.36 | 1.15 (0.85-1.55) |
|  |  |  |  |  |
| **Prasugrel** | 20.0 % (42/210) | 22.6 % (234/1035) | 0.41 | 0.86 (0.59-1.24) |
|  |  |  |  |  |
| **Ticagrelor** | 15.8 % (30/190) | 21.9 % (205/936) | 0.059 | 0.67 (0.44-1.02) |
|  |  |  |  |  |
| **Vitamin-K-Antagonist** | 10.0 % (21/210) | 6.2 % (64/1033) | 0.046 | 1.68 (1.00-2.82) |
|  |  |  |  |  |
| **ACE-inhibitor/ARB** | 48.6 % (102/210) | 57.0 % (589/1034) | 0.026 | 0.71 (0.53-0.96) |
|  |  |  |  |  |
| **Beta-Blocker** | 55.2 % (116/210) | 60.3 % (622/1032) | 0.18 | 0.81 (0.60-1.10) |
|  |  |  |  |  |
| **Diuretic** | 59.0 % (124/210) | 53.7 % (556/1035) | 0.16 | 1.24 (0.92-1.68) |
|  |  |  |  |  |
| **Aldosterone-Antagonist** | 15.7 % (33/210) | 20.6 % (213/1032) | 0.10 | 0.72 (0.48-1.07) |
|  |  |  |  |  |
| **Calcium-Antagonist** | 11.9 % (25/210) | 9.2 % (95/1033) | 0.23 | 1.33 (0.84-2.13) |
|  |  |  |  |  |
| **Statin** | 61.0 % (128/210) | 68.9 % (714/1036) | 0.025 | 0.70 (0.52-0.96) |
|  |  |  |  |  |
| **Moderate and severe bleeding events, % (no./total no.)** | 100.0 % (215/215) | 0.0 % (0/1068) |  |  |
|  |  |  |  |  |
| **Severe/life threatening** | 26.5 % (57/215) | 0.0 % (0/1068) | < 0.001 |  |
|  |  |  |  |  |
| **Moderate** | 78.6 % (169/215) | 0.0 % (0/1068) | < 0.001 |  |
|  |  |  |  |  |
| **30-days events, % (no./total no.)** |  |  |  |  |
|  |  |  |  |  |
| **Death ≤ 30 days** | 47.0 % (101/215) | 43.8 % (467/1067) | 0.39 | 1.14 (0.85-1.53) |
|  |  |  |  |  |
| **Postprocedural death ≤ 30 days** | 46.7 % (100/214) | 40.4 % (407/1007) | 0.089 | 1.29 (0.96-1.74) |
|  |  |  |  |  |
| **Renal replacement therapy ≤ 30 days** | 31.2 % (67/215) | 13.0 % (139/1068) | **< 0.001** | **3.03 (2.16-4.25)** |
|  |  |  |  |  |
| **Events by survivors (≤30)** |  |  |  |  |
|  |  |  |  |  |
| **MI ≤ 30 days** | 2.6 % (3/114) | 2.3 % (14/600) | 0.85 | 1.13 (0.32-4.00) |
|  |  |  |  |  |
| **Stroke ≤ 30 days** | 3.5 % (4/114) | 1.8 % (11/600) | 0.25 | 1.95 (0.61-6.23) |
|  |  |  |  |  |
| **PCI ≤ 30 days** | 7.9 % (9/114) | 4.5 % (27/600) | 0.13 | 1.82 (0.83-3.98) |
|  |  |  |  |  |
| **CABG ≤ 30 days** | 0.9 % (1/114) | 1.3 % (8/600) | 0.69 | 0.65 (0.08-5.29) |
|  |  |  |  |  |
| **1-year events** |  |  |  |  |
|  |  |  |  |  |
| **Death ≤ 365 days** | 59.6 % (127/213) | 51.2 % (545/1065) | **0.024** | **1.41 (1.04-1.90**) |
|  |  |  |  |  |
| **Postprocedural death ≤ 365 days** | 59.4 % (126/212) | 48.3 % (485/1005) | **0.003** | **1.57 (1.16-2.12)** |
|  |  |  |  |  |
| **Events by survivors (≤365)** |  |  |  |  |
|  |  |  |  |  |
| **MI ≤ 365 days** | 4.7 % (4/86) | 4.8 % (25/520) | 0.95 | 0.97 (0.33-2.85) |
|  |  |  |  |  |
| **Stroke ≤ 365 days** | 3.5 % (3/86) | 3.1 % (16/520) | 0.84 | 1.14 (0.32-3.99) |
|  |  |  |  |  |
| **PCI ≤ 365 days** | 29.1 % (25/86) | 26.5 % (138/520) | 0.62 | 1.13 (0.68-1.88) |
|  |  |  |  |  |
| **CABG ≤ 365 days** | 3.5 % (3/86) | 3.5 % (18/520) | 0.99 | 1.01 (0.29-3.50) |
|  |  |  |  |  |
|  |  |  |  |  |

**Legend to Supplemental Table 5:**

This table shows the *logistic* regression model of patients with both moderate and severe bleeding events and patients without bleeding; Data presented are means (± standard deviation, SD), medians (inter quartile range, IQR) or numbers of patients (percentages). BMI, body mass index; PCI, percutaneous coronary intervention; CABG, coronary artery bypass graft; eGFR, estimated glomerular filtration rate; GP IIb/IIIa, glycoprotein IIb/IIIa; UF, unfractionated; LMW, low molecular weight; ACE, angiotensin converting enzyme; ARB, angiotensin receptor blockers; ICU, intensive care unit; LVEF, left ventricular ejection fraction; MI, myocardial infarction; SAPS II, simplified acute physiology score II; OR, odds ratio; CI, confidence interval. P-values: Pearson chi-squared test or Mann-Whitney-Wilcoxon test.

**Supplemental Table 6. *Logistic* *regression model of in-hospital severe and/or moderate bleeding and Cox proportional hazard model for 1-year severe or moderate bleeding***

| **Endpoints** |  | **OR (95%CI)** | **p-value** |
| --- | --- | --- | --- |
| **In- hospital severe/moderate bleeding** | **CULPRIT-SHOCK VS. IABP-SHOCK-II** | 0.76 (0.56-1.02) | 0.063 |
|  |  | **HR (95%CI)** | **p-value** |
| **Severe /moderate bleeding 365- days** | **CULPRIT-SHOCK VS. IABP-SHOCK-II** | 0.76 (0.59-0.99) | **0.039** |

**Legend to Supplemental Table 6:**

This table shows the *logistic* regression model of in- hospital and *a Cox proportional hazard model for* 1- year bleeding events comparing the CULPRIT-SHOCK versus the IABP-SHOCK trial; an unadjusted regression analysis and corresponding odds ratio (OR) or hazard ratio (HR) with 95%-confidence interval (CI) are presented.

**Supplemental Table 7. Modified *logistic* regression *and Cox proportional hazard* model of in- hospital/ 1-year bleeding events**

| **Endpoints** |  | **Unadjusted OR (95%CI)** | **p-value** | **Adjusted OR (95%CI)** | **p-value** |
| --- | --- | --- | --- | --- | --- |
| **In- hospital severe/moderate bleeding** | **Ticagrelor vs. Clopidogrel** | 0.49 (0.28-0.84) | 0.01 | 0.46 (0.23-0.90) | **0.024** |
|  | **Prasugrel vs. Clopidogrel** | 0.72 (0.45-1.17) | 0.18 | 0.79 (0.46-1.35) | 0.39 |
|  | **Ticagrelor vs. Prasugrel** | --- | --- | 0.58 (0.27-1.23) | 0.16 |
|  | **Prasugrel vs. Clopidogrel/Ticagrelor** | --- | --- | 0.81 (0.50-1.32) | 0.39 |
|  | **Ticagrelor vs. Clopidogrel/Prasugrel** | --- | --- | 0.54 (0.30-0.96) | **0.037** |
|  |  | **Unadjusted HR (95%CI)** | **p-value** | **Adjusted HR (95%CI)** | **p-value** |
| **Severe /moderate bleeding 365- days** | **Ticagrelor vs. Clopidogrel** | 0.50 (0.30-0.83) | 0.007 | 0.57 (0.32-1.02) | 0.058 |
|  | **Prasugrel vs. Clopidogrel** | 0.74 (0.49-1.11) | 0.14 | 0.92 (0.60-1.42) | 0.71 |
|  | **Ticagrelor vs. Prasugrel** | --- | --- | 0.62 (0.33-1.17) | 0.14 |
|  | **Prasugrel vs. Clopidogrel/Ticagrelor** | --- | --- | 0.89 (0.60-1.33) | 0.56 |
|  | **Ticagrelor vs. Clopidogrel/Prasugrel** | --- | --- | 0.54 (0.33-0.90) | **0.018** |

**Legend to Supplemental Table 7:**

This table shows the *logistic regression* *for in- hospital* bleedings, *as well as the unadjusted and adjusted Cox regression model of 1- year bleeding events* comparing Prasugrel versus Ticagrelor; an unadjusted and adjusted regression analyses and corresponding odds ratio (OR) or hazard ratio (HR) with 95%-confidence interval (CI) are presented. All variables with p-value <0.05 from the univariate comparison were entered in the multivariable models. For the bleeding model, a logistic regression for the in-hospital events and a Cox proportional hazards regression for the bleeding complications until the end of follow-up was used. Adjusted odds and hazard ratios for bleeding events were calculated with an adjustment for the following variables: Age, gender, previous myocardial infarction, Creatinine on Admission [µmol/l], mechanical ventilation, resuscitation within 24h before randomization, unfractionated heparin as acute drug therapy, serum lactate > 2mmol/l on admission, active assist devices and IABP-SHOCK II vs. CULPRIT-SHOCK. HR, hazard ratio; OR, odds ratio; CI, confidence interval; eGFR, estimated glomerular filtration rate; PCI, percutaneous coronary intervention; CABG, coronary artery bypass graft; SAPS II, simplified acute physiology score II. P-values: Pearson chi-squared test or Mann-Whitney-Wilcoxon test.

**Supplemental Table 8. Baseline characteristics** **of the patients excluded from this analysis**

|  | | **Pts with info to medication** | | **Pts without info** | | **P-value** | | **OR (95%-CI)** | |
| --- | --- | --- | --- | --- | --- | --- | --- | --- | --- |
|  | | | | | | | | | |
| **Number of patients** | | 856 (66.6 %) | | 430 (33.4 %) | |  | |  | |
|  | |  | |  | |  | |  | |
| **Demographics** | |  | |  | |  | |  | |
| Age [year] | | 68 ± 12, N=856 | | 68 ± 12, N=430 | | 0.48 | |  | |
| Median (1. quartile, 3. quartile) | | 70 (59, 77) | | 69 (60, 78) | |  | |  | |
| Female | | 28.4 % (243/856) | | 24.7 % (106/430) | | 0.16 | | 1.21 (0.93-1.58) | |
| Weight [kg] | | 82 ± 15, N=849 | | 82 ± 15, N=419 | | 0.93 | |  | |
| Median (1. quartile, 3. quartile) | | 80 (74, 90) | | 80 (72, 90) | |  | |  | |
| Height [cm] | | 173 ± 9, N=846 | | 173 ± 8, N=412 | | 0.79 | |  | |
| Median (1. quartile, 3. quartile) | | 175 (167, 180) | | 174 (168, 180) | |  | |  | |
| BMI [kg/m] | | 27.7 ± 9.4, N=846 | | 27.6 ± 4.6, N=412 | | 0.71 | |  | |
| Median (1. quartile, 3. quartile) | | 27.2 (24.7, 29.4) | | 26.6 (24.7, 29.4) | |  | |  | |
|  | |  | |  | |  | |  | |
| **Cardiovascular risk factors** | |  | |  | |  | |  | |
| Current smoking | | 32.0 % (270/844) | | 26.4 % (108/409) | | 0.043 | | 1.31 (1.01-1.71) | |
| Hypertension | | 65.4 % (557/852) | | 62.6 % (261/417) | | 0.33 | | 1.13 (0.88-1.44) | |
| Dyslipidemia | | 36.4 % (309/850) | | 35.2 % (146/415) | | 0.68 | | 1.05 (0.82-1.34) | |
| Diabetes mellitus | | 31.2 % (266/852) | | 35.3 % (147/416) | | 0.14 | | 0.83 (0.65-1.06) | |
|  | |  | |  | |  | |  | |
| **Blood pressure at admission** | |  | |  | |  | |  | |
| Heart rate [bpm] | | 89 ± 26, N=821 | | 95 ± 31, N=391 | | 0.027 | |  | |
| Median (1. quartile, 3. quartile) | | 90 (72, 108) | | 93 (75, 110) | |  | |  | |
| Systolic blood pressure [mmHg] | | 98 ± 27, N=793 | | 104 ± 30, N=361 | | 0.003 | |  | |
| Median (1. quartile, 3. quartile) | | 92 (80, 115) | | 100 (82, 120) | |  | |  | |
| Diastolic blood pressure [mmHg] | | 61 ± 19, N=802 | | 64 ± 20, N=358 | | 0.013 | |  | |
| Median (1. quartile, 3. quartile) | | 60 (50, 72) | | 60 (50, 77) | |  | |  | |
| **Medical history: Cardiovascular parameters** | |  | |  | |  | |  | |
| Previous myocardial infarction | | 20.5 % (175/854) | | 16.7 % (70/419) | | 0.11 | | 1.28 (0.95-1.74) | |
| Previous PCI | | 19.8 % (169/854) | | 17.5 % (73/418) | | 0.32 | | 1.17 (0.86-1.58) | |
| Previous CABG surgery | | 4.0 % (34/854) | | 7.3 % (31/423) | | 0.010 | | 0.52 (0.32-0.87) | |
| Previous stroke | | 7.4 % (63/853) | | 7.1 % (30/423) | | 0.85 | | 1.04 (0.67-1.64) | |
| Known peripheral artery disease | | 12.1 % (103/854) | | 11.8 % (50/423) | | 0.90 | | 1.02 (0.71-1.47) | |
| Known renal insufficiency (GFR < 30 ml/min) | | 15.5 % (132/853) | | 9.0 % (38/421) | | 0.001 | | 1.85 (1.26-2.70) | |
|  | |  | |  | |  | |  | |
| Displayed are percentages and numbers or mean and standard deviation P-values: Pearson chi-squared test or Mann-Whitney-Wilcoxon test CI: confidence interval, OR: Odds Ratio | | | | | | | | | |
|  | | **Pts with info to medication** | | **Pts without info** | | **P-value** | | **OR (95%-CI)** | |
|  | | | | | | | | | |
| **Number of patients** | | 856 (66.6 %) | | 430 (33.4 %) | |  | |  | |
|  | |  | |  | |  | |  | |
| **Demographics** | |  | |  | |  | |  | |
| Age [year] | | 68 ± 12, N=856 | | 68 ± 12, N=430 | | 0.48 | |  | |
| Median (1. quartile, 3. quartile) | | 70 (59, 77) | | 69 (60, 78) | |  | |  | |
| Female | | 28.4 % (243/856) | | 24.7 % (106/430) | | 0.16 | | 1.21 (0.93-1.58) | |
| Weight [kg] | | 82 ± 15, N=849 | | 82 ± 15, N=419 | | 0.93 | |  | |
| Median (1. quartile, 3. quartile) | | 80 (74, 90) | | 80 (72, 90) | |  | |  | |
| Height [cm] | | 173 ± 9, N=846 | | 173 ± 8, N=412 | | 0.79 | |  | |
| Median (1. quartile, 3. quartile) | | 175 (167, 180) | | 174 (168, 180) | |  | |  | |
| BMI [kg/m] | | 27.7 ± 9.4, N=846 | | 27.6 ± 4.6, N=412 | | 0.71 | |  | |
| Median (1. quartile, 3. quartile) | | 27.2 (24.7, 29.4) | | 26.6 (24.7, 29.4) | |  | |  | |
|  | |  | |  | |  | |  | |
| **Cardiovascular risk factors** | |  | |  | |  | |  | |
| Current smoking | | 32.0 % (270/844) | | 26.4 % (108/409) | | 0.043 | | 1.31 (1.01-1.71) | |
| Hypertension | | 65.4 % (557/852) | | 62.6 % (261/417) | | 0.33 | | 1.13 (0.88-1.44) | |
| Dyslipidemia | | 36.4 % (309/850) | | 35.2 % (146/415) | | 0.68 | | 1.05 (0.82-1.34) | |
| Diabetes mellitus | | 31.2 % (266/852) | | 35.3 % (147/416) | | 0.14 | | 0.83 (0.65-1.06) | |
|  | |  | |  | |  | |  | |
| **Blood pressure at admission** | |  | |  | |  | |  | |
| Heart rate [bpm] | | 89 ± 26, N=821 | | 95 ± 31, N=391 | | 0.027 | |  | |
| Median (1. quartile, 3. quartile) | | 90 (72, 108) | | 93 (75, 110) | |  | |  | |
| Systolic blood pressure [mmHg] | | 98 ± 27, N=793 | | 104 ± 30, N=361 | | 0.003 | |  | |
| Median (1. quartile, 3. quartile) | | 92 (80, 115) | | 100 (82, 120) | |  | |  | |
| Diastolic blood pressure [mmHg] | | 61 ± 19, N=802 | | 64 ± 20, N=358 | | 0.013 | |  | |
| Median (1. quartile, 3. quartile) | | 60 (50, 72) | | 60 (50, 77) | |  | |  | |
| **Medical history: Cardiovascular parameters** | |  | |  | |  | |  | |
| Previous myocardial infarction | | 20.5 % (175/854) | | 16.7 % (70/419) | | 0.11 | | 1.28 (0.95-1.74) | |
| Previous PCI | | 19.8 % (169/854) | | 17.5 % (73/418) | | 0.32 | | 1.17 (0.86-1.58) | |
| Previous CABG surgery | | 4.0 % (34/854) | | 7.3 % (31/423) | | 0.010 | | 0.52 (0.32-0.87) | |
| Previous stroke | | 7.4 % (63/853) | | 7.1 % (30/423) | | 0.85 | | 1.04 (0.67-1.64) | |
| Known peripheral artery disease | | 12.1 % (103/854) | | 11.8 % (50/423) | | 0.90 | | 1.02 (0.71-1.47) | |
| Known renal insufficiency (GFR < 30 ml/min) | | 15.5 % (132/853) | | 9.0 % (38/421) | | 0.001 | | 1.85 (1.26-2.70) | |
|  | |  | |  | |  | |  | |
| Displayed are percentages and numbers or mean and standard deviation P-values: Pearson chi-squared test or Mann-Whitney-Wilcoxon test CI: confidence interval, OR: Odds Ratio | | | | | | | | | |

**Supplemental Table 9. Clinical parameters of the patients excluded from this analysis**

|  | **Pts with info to medication** | **Pts without info** | **P-value** | **OR (95%-CI)** |
| --- | --- | --- | --- | --- |
|  | | | | |
| **Number of patients** | 856 (66.6 %) | 430 (33.4 %) |  |  |
|  |  |  |  |  |
| **Chronic drug therapy** |  |  |  |  |
| Aspirin | 40.1 % (311/776) | 40.2 % (136/338) | 0.96 | 0.99 (0.77-1.29) |
| Clopidogrel | 11.4 % (88/771) | 8.7 % (29/334) | 0.18 | 1.36 (0.87-2.11) |
| Prasugrel | 0.5 % (4/771) | 3.3 % (11/333) | < 0.001 | 0.15 (0.05-0.48) |
| Ticagrelor | 2.9 % (18/631) | 1.3 % (4/311) | 0.13 | 2.25 (0.76-6.72) |
| Vitamin-K-Antagonists | 4.9 % (38/768) | 5.1 % (17/335) | 0.93 | 0.97 (0.54-1.75) |
| Beta-Blocker | 38.9 % (298/766) | 38.7 % (129/333) | 0.96 | 1.01 (0.77-1.31) |
| ACE-Inhibitor/AT-II-Antagonist | 43.4 % (333/768) | 46.7 % (155/332) | 0.31 | 0.87 (0.68-1.13) |
| Statin | 29.6 % (227/767) | 37.0 % (123/332) | 0.015 | 0.71 (0.54-0.94) |
|  |  |  |  |  |
| **Clinical and laboratory parameters** |  |  |  |  |
| Altered mental status | 70.7 % (604/854) | 71.2 % (304/427) | 0.86 | 0.98 (0.76-1.26) |
| Cold, clammy skin and extremities | 79.1 % (674/852) | 70.7 % (297/420) | < 0.001 | 1.57 (1.20-2.05) |
| Oliguria (≤ 30ml/h) | 27.9 % (237/848) | 30.4 % (125/411) | 0.36 | 0.89 (0.69-1.15) |
| pH < 7.36 | 60.5 % (512/846) | 63.5 % (264/416) | 0.31 | 0.88 (0.69-1.12) |
| Serum-lactate > 2mmol/l | 70.0 % (593/847) | 70.0 % (291/416) | 0.98 | 1.00 (0.78-1.30) |
|  |  |  |  |  |
| Mechanical ventilation | 54.0 % (462/855) | 67.2 % (287/427) | < 0.001 | 0.57 (0.45-0.73) |
| Resuscitation within 24h before randomization | 44.0 % (376/855) | 60.7 % (260/428) | < 0.001 | 0.51 (0.40-0.64) |
|  |  |  |  |  |
| **ECG pre PCI** |  |  |  |  |
| Pacemaker rhythm | 2.6 % (22/851) | 2.4 % (10/412) | 0.87 | 1.07 (0.50-2.27) |
| Left bundle branch block (LBBB) | 13.0 % (110/849) | 16.0 % (66/412) | 0.14 | 0.78 (0.56-1.09) |
| ST-segment elevation | 65.6 % (557/849) | 56.2 % (231/411) | 0.001 | 1.49 (1.17-1.89) |
| Anterior infarction | 40.6 % (343/845) | 32.0 % (131/410) | 0.003 | 1.46 (1.13-1.87) |
| Non-anterior infarction | 30.5 % (258/845) | 28.0 % (115/410) | 0.37 | 1.13 (0.87-1.46) |
| Other Rythm | 10.9 % (93/853) | 15.2 % (63/415) | 0.030 | 0.68 (0.48-0.96) |
|  |  |  |  |  |
| Displayed are percentages and numbers P-values: Pearson chi-squared test CI: confidence interval, OR: Odds Ratio | | | | |

**Supplemental Table 10. Baseline laboratory parameters of the patients excluded from this analysis**

|  | **Pts with info to medication** | **Pts without info** | **P-value** | **Effects** |
| --- | --- | --- | --- | --- |
|  | | | | |
| **Number of patients** | 856 (66.6 %) | 430 (33.4 %) |  |  |
|  |  |  |  |  |
| **Laboratory at admission** |  |  |  |  |
| Glucose on Admission [mmol/l] | 16.51 ± 49.59, N=715 | 14.17 ± 9.69, N=321 | 0.013 |  |
| Median (1. quartile, 3. quartile) | 11.50 (8.04, 16.50) | 12.50 (8.99, 17.40) |  |  |
|  |  |  |  |  |
| Creatinine on Admission [umol/l] | 130.90 ± 85.48, N=838 | 161.62 ± 616.89, N=397 | 0.94 |  |
| Median (1. quartile, 3. quartile) | 112.63 (92.00, 145.00) | 112.27 (91.00, 144.09) |  |  |
|  |  |  |  |  |
| Hemoglobin [mmol/l] | 8.69 ± 6.33, N=839 | 10.46 ± 39.18, N=409 | 0.11 |  |
| Median (1. quartile, 3. quartile) | 8.32 (7.40, 9.12) | 8.25 (7.20, 9.00) |  |  |
|  |  |  |  |  |
| GFR nach Cockroft und Gault Formel [ml/min] | 62.68 ± 32.40, N=498 | 100.31 ± 459.97, N=317 | 0.47 |  |
| Median (1. quartile, 3. quartile) | 58.15 (40.25, 78.93) | 59.78 (43.49, 78.48) |  |  |
|  |  |  |  |  |
| Body Temperature | 36.23 ± 1.48, N=774 | 35.99 ± 1.66, N=349 | 0.060 |  |
| Median (1. quartile, 3. quartile) | 36.50 (35.20, 37.30) | 36.30 (35.00, 37.20) |  |  |
|  |  |  |  |  |
| SAPS II Score | 50.84 ± 23.80, N=808 | 54.95 ± 23.98, N=374 | 0.004 |  |
| Median (1. quartile, 3. quartile) | 50.50 (35.00, 68.00) | 56.00 (37.00, 73.00) |  |  |
|  |  |  |  |  |
| **LVEF measurements** |  |  |  |  |
| Acute LVEF [%] | 35 ± 13, N=476 | 35 ± 14, N=177 | 0.80 |  |
| Median (1. quartile, 3. quartile) | 35 (25, 44) | 35 (25, 45) |  |  |
|  |  |  |  |  |
| **Hemodynamic measurements before PCI** |  |  |  |  |
| Heart rate [bpm] | 88.94 ± 24.92, N=847 | 92.14 ± 28.43, N=421 | 0.15 |  |
| Diastolic blood pressure [mmHg] | 58.06 ± 16.42, N=847 | 58.44 ± 18.12, N=417 | 0.81 |  |
| Systolic blood pressure [mmHg] | 95.39 ± 25.96, N=847 | 97.97 ± 27.57, N=418 | 0.18 |  |
|  |  |  |  |  |
| **Hemodynamic measurements after PCI** |  |  |  |  |
| Heart rate after PCI [/min] | 89.61 ± 22.91, N=831 | 88.81 ± 24.02, N=385 | 0.46 |  |
| Diastolic blood pressure [mmHg] | 61.35 ± 16.32, N=830 | 62.99 ± 16.77, N=380 | 0.034 |  |
| Systolic blood pressure [mmHg] | 106.24 ± 25.97, N=830 | 107.90 ± 26.46, N=383 | 0.28 |  |
|  |  |  |  |  |
| Displayed are mean and standard deviation or percentages and numbers P-values: Mann-Whitney-Wilcoxon test | | | | |

**Supplemental Table 11. Serum lactate of the patients excluded from this analysis**

|  | **Pts with info to medication** | **Pts without info** | **P-value** | **Effects** |
| --- | --- | --- | --- | --- |
|  | | | | |
| **Number of patients** | 856 (66.6 %) | 430 (33.4 %) |  |  |
|  |  |  |  |  |
| **Serum Lactate** |  |  |  |  |
|  |  |  |  |  |
| Serum Lactat pre PCI [mmol/l] | 5.40 ± 4.81, N=693 | 7.17 ± 10.06, N=299 | < 0.001 |  |
| Median (1. quartile, 3. quartile) | 3.90 (2.13, 7.40) | 5.10 (2.66, 8.20) |  |  |
|  |  |  |  |  |
| 8h after PCI [mmol/l] | 3.98 ± 5.56, N=711 | 4.52 ± 5.65, N=316 | 0.011 |  |
| Median (1. quartile, 3. quartile) | 2.10 (1.30, 4.70) | 2.60 (1.50, 5.25) |  |  |
|  |  |  |  |  |
| 16h after PCI [mmol/l] | 3.12 ± 3.39, N=674 | 3.67 ± 5.22, N=289 | 0.25 |  |
| Median (1. quartile, 3. quartile) | 1.80 (1.20, 3.40) | 2.00 (1.20, 4.00) |  |  |
|  |  |  |  |  |
| 24h after PCI [mmol/l] | 2.66 ± 2.83, N=636 | 3.04 ± 3.63, N=268 | 0.090 |  |
| Median (1. quartile, 3. quartile) | 1.70 (1.14, 2.80) | 1.80 (1.20, 3.80) |  |  |
|  |  |  |  |  |
| 32h after PCI [mmol/l] | 2.39 ± 2.73, N=605 | 2.64 ± 3.10, N=251 | 0.100 |  |
| Median (1. quartile, 3. quartile) | 1.44 (1.10, 2.30) | 1.60 (1.10, 2.90) |  |  |
|  |  |  |  |  |
| 40h after PCI [mmol/l] | 2.36 ± 6.63, N=572 | 2.24 ± 2.51, N=239 | 0.62 |  |
| Median (1. quartile, 3. quartile) | 1.40 (1.00, 2.10) | 1.40 (1.00, 2.40) |  |  |
|  |  |  |  |  |
| 48h after PCI [mmol/l] | 2.07 ± 4.53, N=554 | 1.96 ± 1.90, N=228 | 0.43 |  |
| Median (1. quartile, 3. quartile) | 1.40 (1.00, 1.90) | 1.40 (1.00, 2.10) |  |  |
|  |  |  |  |  |
| Displayed are mean and standard deviation or percentages and numbers P-values: Mann-Whitney-Wilcoxon test | | | | |

**Supplemental Table 12. Culprit lesion of the patients excluded from this analysis**

|  | **Pts with info to medication** | **Pts without info** | **P-value** | **OR (95%-CI)** |
| --- | --- | --- | --- | --- |
|  | | | | |
| **No. of diseased vessels** |  |  |  |  |
| Single vessel disease | 12.7 % (108/852) | 5.4 % (23/423) |  | 2.52 (1.58-4.02) |
| Double vessel disease | 31.8 % (271/852) | 31.0 % (131/423) |  | 1.04 (0.81-1.34) |
| Triple vessel disease | 55.5 % (473/852) | 63.6 % (269/423) |  | 0.71 (0.56-0.91) |
|  |  |  |  |  |
| **Artery with culprit lesion** |  |  |  |  |
| RCA | 26.8 % (228/852) | 27.2 % (115/423) |  | 0.98 (0.75-1.27) |
| Left main | 8.1 % (69/852) | 9.0 % (38/423) |  | 0.89 (0.59-1.35) |
| LAD | 43.0 % (366/852) | 41.6 % (176/423) |  | 1.06 (0.83-1.34) |
| RCX | 20.5 % (175/852) | 19.6 % (83/423) |  | 1.06 (0.79-1.42) |
| Bypass | 1.4 % (12/851) | 2.4 % (10/423) |  | 0.59 (0.25-1.38) |
|  |  |  |  |  |
| Stent implanted in lesions | 94.8 % (799/843) | 93.5 % (389/416) | 0.36 | 1.26 (0.77-2.07) |
| Bare metal stent culprit lesion | 36.0 % (288/799) | 13.9 % (54/389) | < 0.001 | 3.50 (2.53-4.82) |
| Drug-eluting stent culprit lesion | 65.6 % (524/799) | 86.9 % (338/389) | < 0.001 | 0.29 (0.21-0.40) |
| Bioabsorbable scaffold culprit lesion | 0.6 % (2/341) | 1.0 % (3/309) | 0.58 | 0.60 (0.10-3.63) |
|  |  |  |  |  |
| Manual thrombectomy before stenting | 22.2 % (190/855) | 13.5 % (58/430) | < 0.001 | 1.83 (1.33-2.52) |
| Manual thrombectomy after stenting | 2.3 % (20/855) | 2.3 % (10/430) | 0.99 | 1.01 (0.47-2.17) |
|  |  |  |  |  |
| **TIMI-flow pre PCI culprit lesion** |  |  | 0.038 |  |
| TIMI-flow 0 | 59.0 % (495/839) | 53.5 % (220/411) |  | 1.25 (0.98-1.58) |
| TIMI-flow I | 11.8 % (99/839) | 12.9 % (53/411) |  | 0.90 (0.63-1.29) |
| TIMI-flow II | 14.9 % (125/839) | 14.4 % (59/411) |  | 1.04 (0.75-1.46) |
| TIMI-flow III | 14.3 % (120/839) | 19.2 % (79/411) |  | 0.70 (0.51-0.96) |
|  |  |  |  |  |
| **TIMI-flow post PCI culprit lesion** |  |  | 0.10 |  |
| TIMI-flow 0 | 3.9 % (33/840) | 5.8 % (24/414) |  | 0.66 (0.39-1.14) |
| TIMI-flow I | 3.6 % (30/840) | 3.4 % (14/414) |  | 1.06 (0.55-2.02) |
| TIMI-flow II | 7.5 % (63/840) | 9.4 % (39/414) |  | 0.78 (0.51-1.18) |
| TIMI-flow III | 85.0 % (714/840) | 81.4 % (337/414) |  | 1.29 (0.95-1.77) |
|  |  |  |  |  |
| Stent thrombosis | 1.3 % (11/855) | 3.5 % (15/430) | 0.008 | 0.36 (0.16-0.79) |
|  |  |  |  |  |
| Displayed are percentages and numbers P-values: Pearson chi-squared test or Mann-Whitney-Wilcoxon test CI: confidence interval, OR: Odds Ratio | | | | |

**Supplemental Table 13. Intensive care treatment of the patients excluded from this analysis**

|  | **Pts with info to medication** | **Pts without info** | **P-value** | **OR (95%-CI)** |
| --- | --- | --- | --- | --- |
|  | | | | |
| **Number of patients** | 856 (66.6 %) | 430 (33.4 %) |  |  |
|  |  |  |  |  |
| Immediate PCI of additional lesions | 37.7 % (318/844) | 49.4 % (206/417) | < 0.001 | 0.62 (0.49-0.78) |
|  |  |  |  |  |
| Indication for immediate CABG | 1.2 % (9/774) | 0.5 % (2/404) | 0.26 | 2.36 (0.51-11.00) |
|  |  |  |  |  |
|  |  |  |  |  |
| Mild induced Hypothermia | 30.9 % (264/855) | 37.8 % (162/429) | 0.013 | 0.74 (0.58-0.94) |
|  |  |  |  |  |
| Catecholamine requirement | 89.0 % (761/855) | 95.3 % (408/428) | < 0.001 | 0.40 (0.24-0.65) |
| Epinephrine | 38.1 % (290/761) | 44.1 % (180/408) | 0.046 | 0.78 (0.61-1.00) |
| Norepinephrine | 86.5 % (658/761) | 92.9 % (379/408) | < 0.001 | 0.49 (0.32-0.75) |
| Dobutamine | 58.6 % (446/761) | 59.3 % (242/408) | 0.81 | 0.97 (0.76-1.24) |
| Dopamine | 9.5 % (72/761) | 2.0 % (8/408) | < 0.001 | 5.22 (2.49-10.96) |
|  |  |  |  |  |
| **Mechanical ventilation** | 77.1 % (659/855) | 90.7 % (388/428) | < 0.001 | 0.35 (0.24-0.50) |
| Invasive | 94.1 % (620/659) | 92.3 % (358/388) | 0.25 | 1.33 (0.81-2.18) |
| Non-invasive | 16.1 % (106/659) | 20.1 % (78/388) | 0.099 | 0.76 (0.55-1.05) |
|  |  |  |  |  |
| **Duration of all patients** |  |  |  |  |
| Duration of mechanical ventilation [day] | 3.0 (1.0, 8.0) | 3.0 (1.0, 8.0) | 0.16 |  |
| Duration of ICU treatment [day] | 5.0 (2.0, 11.0) | 5.5 (2.0, 13.0) | 0.62 |  |
| Duration of catecholamine [day] | 3.0 (1.0, 5.0) | 2.0 (1.0, 5.0) | 0.67 |  |
| Days to hemodynamic stabilization [day] | 3.0 (1.0, 6.0) | 3.0 (1.0, 6.0) | 0.96 |  |
|  |  |  |  |  |
| Displayed are percentages and numbers or median and quartiles P-values: Pearson chi-squared test or Mann-Whitney-Wilcoxon test CI: confidence interval, OR: Odds Ratio | | | | |

**Supplemental Table 14. Detailed acute drug therapy and interventions of the patients excluded from this analysis**

|  | **Pts with info to medication** | **Pts without info** | **P-value** | **OR (95%-CI)** |
| --- | --- | --- | --- | --- |
|  | | | | |
| **Number of patients** | 856 (66.6 %) | 430 (33.4 %) |  |  |
| **Medication: Acute drug therapy*** |  |  |  |  |
| Aspirin | 91.2 % (781/856) | 61.8 % (264/427) | < 0.001 | 6.43 (4.73-8.74) |
| Clopidogrel | 59.2 % (507/856) | 4.7 % (20/427) | < 0.001 | 29.56 (18.49-47.26) |
| Prasugrel | 20.8 % (178/856) | 3.5 % (15/427) | < 0.001 | 7.21 (4.20-12.39) |
| Ticagrelor | 23.8 % (171/718) | 2.2 % (9/406) | < 0.001 | 13.79 (6.97-27.29) |
| GP IIb/IIIa-Inhibitors | 36.4 % (312/856) | 27.2 % (116/427) | < 0.001 | 1.54 (1.19-1.98) |
| UF Heparin | 89.3 % (764/856) | 80.3 % (343/427) | < 0.001 | 2.03 (1.47-2.81) |
| LMW Heparin | 8.2 % (70/856) | 11.5 % (49/427) | 0.055 | 0.69 (0.47-1.01) |
| Bivalirudin | 7.8 % (67/856) | 8.9 % (38/427) | 0.51 | 0.87 (0.57-1.32) |
|  |  |  |  |  |
| **Medication at discharge** |  |  |  |  |
|  |  |  |  |  |
| **Anticoagulative/Antithrombotic Medication** |  |  |  |  |
| Aspirin | 89.2 % (742/832) | 74.9 % (311/415) | < 0.001 | 2.76 (2.02-3.77) |
| Clopidogrel | 53.2 % (443/832) | 31.8 % (132/415) | < 0.001 | 2.44 (1.91-3.13) |
| Prasugrel | 22.4 % (186/831) | 21.7 % (90/415) | 0.78 | 1.04 (0.78-1.38) |
| Ticagrelor | 20.2 % (147/727) | 22.3 % (89/400) | 0.42 | 0.89 (0.66-1.19) |
| Vitamin-K-Antagonists | 6.4 % (53/830) | 7.7 % (32/414) | 0.38 | 0.81 (0.52-1.28) |
| ACE-Inhibitor/AT-II-Antagonist | 59.9 % (497/830) | 47.0 % (195/415) | < 0.001 | 1.68 (1.33-2.14) |
| Beta-Blocker | 62.8 % (520/828) | 52.8 % (219/415) | < 0.001 | 1.51 (1.19-1.92) |
| Diuretics | 59.8 % (497/831) | 44.1 % (183/415) | < 0.001 | 1.89 (1.49-2.39) |
| Aldosterone-Antagonist | 22.7 % (188/828) | 14.2 % (59/415) | < 0.001 | 1.77 (1.29-2.44) |
| Calcium-Antagonist | 10.3 % (85/829) | 8.7 % (36/415) | 0.38 | 1.20 (0.80-1.81) |
| Statins | 73.3 % (610/832) | 56.1 % (233/415) | < 0.001 | 2.15 (1.68-2.75) |
|  |  |  |  |  |
| **Moderate and severe bleeding** | 15.9 % (136/855) | 18.4 % (79/429) | 0.26 | 0.84 (0.62-1.14) |
| Severe/lifethreatening | 4.3 % (37/855) | 4.7 % (20/429) | 0.78 | 0.93 (0.53-1.61) |
| Moderate | 12.9 % (110/855) | 13.8 % (59/429) | 0.66 | 0.93 (0.66-1.30) |
|  |  |  |  |  |
| Displayed are percentages and numbers P-values: Pearson chi-squared test CI: confidence interval, OR: Odds Ratio | | | | |

**Supplemental Table 15. Detailed acute drug therapy and interventions of the patients excluded from this analysis**

|  | **Pts with info to medication** | **Pts without info** | **P-value** | **OR (95%-CI)** |
| --- | --- | --- | --- | --- |
|  | | | | |
| **Number of patients** | 856 (66.6 %) | 430 (33.4 %) |  |  |
|  |  |  |  |  |
| **30-days events** |  |  |  |  |
| Death ≤ 30 days | 40.6 % (347/855) | 51.6 % (222/430) | < 0.001 | 0.64 (0.51-0.81) |
| Postprocedural death ≤ 30 days | 38.9 % (323/831) | 46.5 % (181/389) | 0.011 | 0.73 (0.57-0.93) |
|  |  |  |  |  |
| Renal replacement therapy ≤ 30 days | 17.3 % (148/856) | 13.5 % (58/430) | 0.080 | 1.34 (0.97-1.86) |
|  |  |  |  |  |
| **Events by survivors (≤30)** |  |  |  |  |
| MI ≤ 30 days | 2.0 % (10/508) | 3.4 % (7/208) | 0.27 | 0.58 (0.22-1.54) |
| Stroke ≤ 30 days | 1.8 % (9/508) | 2.9 % (6/208) | 0.35 | 0.61 (0.21-1.73) |
| PCI ≤ 30 days | 6.1 % (31/508) | 2.4 % (5/208) | 0.040 | 2.64 (1.01-6.88) |
| CABG ≤ 30 days | 1.4 % (7/508) | 1.0 % (2/208) | 0.65 | 1.44 (0.30-6.99) |
|  |  |  |  |  |
| **1-year events** |  |  |  |  |
| Death ≤ 365 days | 50.0 % (426/852) | 57.6 % (247/429) | 0.010 | 0.74 (0.58-0.93) |
| Postprocedural death ≤ 365 days | 48.6 % (402/828) | 53.1 % (206/388) | 0.14 | 0.83 (0.65-1.06) |
|  |  |  |  |  |
| **Events by survivors (≤365)** |  |  |  |  |
| MI ≤ 365 days | 4.9 % (21/426) | 4.4 % (8/182) | 0.78 | 1.13 (0.49-2.60) |
| Stroke ≤ 365 days | 3.1 % (13/426) | 3.3 % (6/182) | 0.87 | 0.92 (0.35-2.47) |
| PCI ≤ 365 days | 24.6 % (105/426) | 31.9 % (58/182) | 0.066 | 0.70 (0.48-1.02) |
| CABG ≤ 365 days | 4.0 % (17/426) | 2.2 % (4/182) | 0.27 | 1.85 (0.61-5.58) |
|  |  |  |  |  |
| Moderate and severe bleeding ≤ 365 | 13.1 % (56/426) | 23.1 % (42/182) | 0.002 | 0.50 (0.32-0.79) |
| Severe bleeding ≤ 365 days | 2.6 % (11/426) | 4.4 % (8/182) | 0.24 | 0.58 (0.23-1.46) |
| Moderate bleeding ≤ 365 days | 11.5 % (49/426) | 18.7 % (34/182) | 0.018 | 0.57 (0.35-0.91) |
|  |  |  |  |  |
| Displayed are percentages and numbers P-values: Pearson chi-squared test CI: confidence interval, OR: Odds Ratio | | | | |

**Supplemental Table 16. Clinical outcome at follow-up of the patients excluded from this analysis**

|  | **Pts with info to medication** | **Pts without info** | **P-value** | **OR (95%-CI)** |
| --- | --- | --- | --- | --- |
|  | | | | |
| **Number of patients** | 856 (66.6 %) | 430 (33.4 %) |  |  |
|  |  |  |  |  |
| **30-days events** |  |  |  |  |
| Death ≤ 30 days | 40.6 % (347/855) | 51.6 % (222/430) | < 0.001 | 0.64 (0.51-0.81) |
| Postprocedural death ≤ 30 days | 38.9 % (323/831) | 46.5 % (181/389) | 0.011 | 0.73 (0.57-0.93) |
|  |  |  |  |  |
| Renal replacement therapy ≤ 30 days | 17.3 % (148/856) | 13.5 % (58/430) | 0.080 | 1.34 (0.97-1.86) |
|  |  |  |  |  |
| **Events by survivors (≤30)** |  |  |  |  |
| MI ≤ 30 days | 2.0 % (10/508) | 3.4 % (7/208) | 0.27 | 0.58 (0.22-1.54) |
| Stroke ≤ 30 days | 1.8 % (9/508) | 2.9 % (6/208) | 0.35 | 0.61 (0.21-1.73) |
| PCI ≤ 30 days | 6.1 % (31/508) | 2.4 % (5/208) | 0.040 | 2.64 (1.01-6.88) |
| CABG ≤ 30 days | 1.4 % (7/508) | 1.0 % (2/208) | 0.65 | 1.44 (0.30-6.99) |
|  |  |  |  |  |
| **1-year events** |  |  |  |  |
| Death ≤ 365 days | 50.0 % (426/852) | 57.6 % (247/429) | 0.010 | 0.74 (0.58-0.93) |
| Postprocedural death ≤ 365 days | 48.6 % (402/828) | 53.1 % (206/388) | 0.14 | 0.83 (0.65-1.06) |
|  |  |  |  |  |
| **Events by survivors (≤365)** |  |  |  |  |
| MI ≤ 365 days | 4.9 % (21/426) | 4.4 % (8/182) | 0.78 | 1.13 (0.49-2.60) |
| Stroke ≤ 365 days | 3.1 % (13/426) | 3.3 % (6/182) | 0.87 | 0.92 (0.35-2.47) |
| PCI ≤ 365 days | 24.6 % (105/426) | 31.9 % (58/182) | 0.066 | 0.70 (0.48-1.02) |
| CABG ≤ 365 days | 4.0 % (17/426) | 2.2 % (4/182) | 0.27 | 1.85 (0.61-5.58) |
|  |  |  |  |  |
| Moderate and severe bleeding ≤ 365 | 13.1 % (56/426) | 23.1 % (42/182) | 0.002 | 0.50 (0.32-0.79) |
| Severe bleeding ≤ 365 days | 2.6 % (11/426) | 4.4 % (8/182) | 0.24 | 0.58 (0.23-1.46) |
| Moderate bleeding ≤ 365 days | 11.5 % (49/426) | 18.7 % (34/182) | 0.018 | 0.57 (0.35-0.91) |
|  |  |  |  |  |
| Displayed are percentages and numbers P-values: Pearson chi-squared test CI: confidence interval, OR: Odds Ratio | | | | |

**Legend to Supplemental Tables 8 to 16:**

These tables show various characteristics of the patients excluded from this analysis; Data presented are means (± standard deviation, SD), medians (inter quartile range, IQR) or numbers of patients (percentages). BMI, body mass index; PCI, percutaneous coronary intervention; CABG, coronary artery bypass graft; eGFR, estimated glomerular filtration rate; GP IIb/IIIa, glycoprotein IIb/IIIa; UF, unfractionated; LMW, low molecular weight; ACE, angiotensin converting enzyme; ARB, angiotensin receptor blockers; ICU, intensive care unit; LVEF, left ventricular ejection fraction; MI, myocardial infarction; SAPS II, simplified acute physiology score II; OR, odds ratio; CI, confidence interval. P-values: Pearson chi-squared test or Mann-Whitney-Wilcoxon test.
